# Direct cell-to-cell transmission of retrotransposons

**DOI:** 10.1101/2025.03.14.642691

**Published:** 2025-03-16

**Authors:** Maya Voichek, Andreas Bernhard, Maria Novatchkova, Dominik Handler, Paul Möseneder, Baptiste Rafanel, Peter Duchek, Kirsten-André Senti, Julius Brennecke

**Affiliations:** 1 Institute of Molecular Biotechnology of the Austrian Academy of Sciences (IMBA), Vienna BioCenter (VBC); Dr. Bohr-Gasse 3, 1030 Vienna, Austria.; 2 Vienna BioCenter PhD Program, Doctoral School of the University of Vienna and Medical University of Vienna, Vienna, Austria.

## Abstract

Transposable elements are abundant in host genomes but are generally considered to be confined to the cell in which they are expressed, with the notable exception of endogenous retroviruses. Here, we identify a group of LTR retrotransposons that infect the germline from somatic cells within the *Drosophila* ovary, despite lacking the fusogenic Envelope protein typically required for retroviral entry. Instead, these elements encode a short transmembrane protein, sORF2, with structural features reminiscent of viral cell-cell fusogens. Through genetics, imaging, and electron microscopy, we show that sORF2 localizes to invasive somatic protrusions, enabling the direct transfer of retrotransposon capsids into the oocyte. Remarkably, sORF2-like proteins are widespread among insect retrotransposons and also occur in piscine nackednaviruses and avian picornaviruses. These findings reveal a noncanonical, Envelope-independent transmission mechanism shared by retrotransposons and non-enveloped viruses, offering important insights into host-pathogen evolution and soma-germline interactions.

## INTRODUCTION

Viruses are “master hackers” of cell biology and understanding the molecular determinants that underlie their life cycles, particularly cell-to-cell transmission, has important medical and scientific implications. While much of what we know about viral entry and exit mechanisms comes from studying modern human pathogens, these represent only a small fraction of the viral realm. Throughout evolution, most virus-host battles have been won or lost without leaving a trace, but vertebrate endogenous retroviruses (ERVs) are a fascinating exception. These genomic fossils are thought to have arisen from rare germline infections of exogenous retroviruses that led to the integration of the viral genome into the host genome and are thus often grouped together with transposable elements (TEs), specifically long terminal repeats (LTR) retrotransposons (Johnson, 2019). The repertoire of ERVs present in a host genome hence captures a “snapshot” of past infections, providing a unique opportunity to study the molecular basis of viral infectivity. However, ERVs degenerate over time by accumulating mutations due to powerful repression mechanisms against selfish genetic elements in the host germline (Levin & Moran, 2011). Together with the high copy number of ERVs in vertebrate genomes, studying their biology and in particular their infectivity poses significant challenges.

The fruit fly *Drosophila melanogaster* offers an alternative promising model for studying endogenous retroviruses in the context of germline biology. Its genome harbors a wealth of relatively young, diverse and, above all, active ERVs (Kaminker et al., 2002; Kapitonov & Jurka, 2003). While insect ERVs (family *Metaviridae*) are phylogenetically distinct from vertebrate ERVs (family *Retroviridae*), some share key structural and functional features, including the canonical retroviral genome organization with *gag*, *pol* and *env* genes flanked by two LTRs (fig. S1A) (Llorens et al., 2020; Malik et al., 2000). Under permissive conditions, enveloped insect-ERVs such as *Gypsy* or *ZAM* are expressed in somatic cells of the ovary and infect the neighboring oocyte as retroviral particles (A. Kim et al., 1994; Leblanc et al., 2000; Song et al., 1994; Yoth et al., 2023). Similar to vertebrate retroviruses, this cell-to-cell infectivity is thought to require an intact *envelope* (*env*) gene, which encodes a fusogenic protein mediating the virus-cell membranes fusion (Misseri et al., 2004; Pélisson et al., 1994). In contrast, many other LTR retrotransposons in the *Drosophila* genome lack a functional *env* gene and are expressed in the germline lineage like other TEs (Bourque et al., 2018). Both insect ERVs and LTR retrotransposons are suppressed by the host defense system, the PIWI/piRNA pathway, which is active in the gonad (Malone & Hannon, 2009; Senti et al., 2023). Upon genetic disruption of the piRNA pathway, active insect ERVs and LTR retrotransposons are expressed, creating an opportunity to investigate their native biology. Utilizing the rich toolkit of *Drosophila* genetics, we set out to pursue the possibility of novel features that confer infectivity to LTR retrotransposons.

In this study, we uncover an *env*-independent transmission mechanism that enables a group of LTR retrotransposons, the MDG1 elements, to directly enter the well-protected oocyte from neighboring somatic cells. This discovery highlights an evolutionary innovation by which an ancestral LTR retrotransposon has gained viral-like cell-to-cell transmission capability, further blurring the conventional boundaries between transposons and viruses (Widen et al., 2023). Remarkably, we find that similar *env*-independent infectivity strategies are prevalent among non-enveloped viruses infecting a broad spectrum of hosts.

## RESULTS

### LTR retrotransposons of the MDG1 group behave as retroviruses

**Figure 1:**
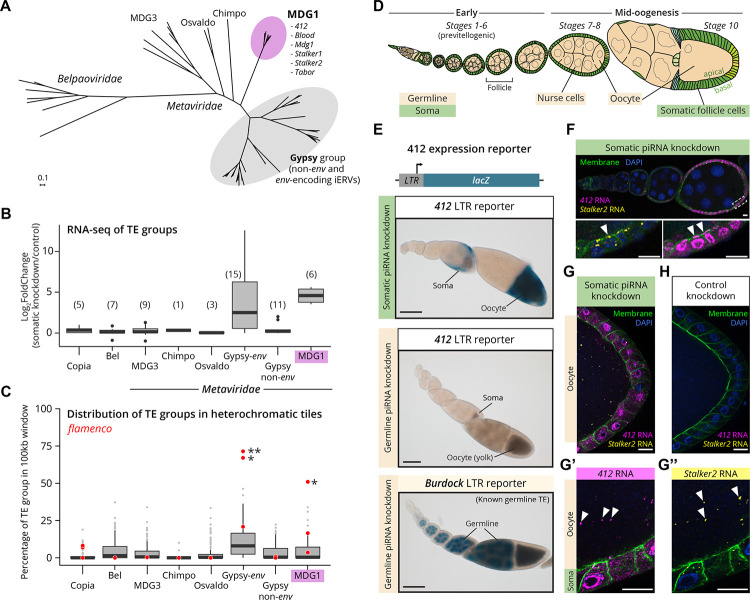
Somatically expressed *412* and *Stalker2* RNA transmits into the oocyte. **(A)** Phylogenetic tree of *Metaviridae* in *D. melanogaster* based on a multiple sequence alignment of the consensus *pol* region, rooted with *Belpaoviridae* as an outgroup. The Gypsy group (grey circle) includes both *env*-encoding insect ERVs and LTR retrotransposons lacking an *env* gene. The MDG1 group is highlighted in magenta. Scale bar: substitutions per site. **(B)** Expression fold change of transposable element (TE) groups measured by RNA-seq of ovaries upon somatic piRNA pathway knockdown (*Tj*>*Gal4, vret-RNAi*) compared to control (*Tj*>*Gal4, arr2-RNAi*) (n=3). The number of TEs in each group is indicated in brackets. **(C)** TE group distribution within TE-enriched heterochromatic tiles (100-kb genomic regions with >50% transposon content). Red datapoints represent *flamenco* tiles. Asterisks indicate significant deviation from the background distribution (MDG1 *p*-value = 0.0145). For (B-C) Gypsy-*env* refers to *env*-encoding insect ERVs, and Gypsy non-*env* refers to LTR retrotransposons without an *env* gene. Boxplots in (B-C) present the median (center line), IQR (box bounds), min and max within 1.5 IQR (whiskers), and outliers (points beyond whiskers). **(D)** Schematic of a *Drosophila* ovariole, showing sequential progression of follicles through the developmental stages of oogenesis in the adult ovary. Somatic follicle cells (green) are separate from the germline (beige), which is comprised of the oocyte and nurse cells. **(E)** Transcriptional *LTR-lacZ* reporters for *412* under different genetic conditions, (top) somatic piRNA pathway knockdown (*Tj*>*Gal4, vret-RNAi*); (middle) germline piRNA pathway knockdown (*MTD*>*Gal4, aub-RNAi, ago3-RNAi*); (bottom) *LTR-lacZ* reporter for germline-expressed *Burdock* TE under germline piRNA pathway knockdown (*MTD*>*Gal4, aub-RNAi, ago3-RNAi*). Scale bar: 100 μm. **(F-H)** Dual smFISH staining of early (F) and mid-oogenesis follicles (G-H) for LTR retrotransposons *412* (magenta) and *Stalker2* (yellow) RNA. Panels **(F)** and **(G, G’, G”)** show somatic piRNA pathway knockdown (*Tj*>*Gal4, vret-RNAi*) while **(H)** shows the control knockdown for an unrelated gene (*Tj*>*Gal4, arr2-RNAi*). Panel (F) bottom shows zoom-in of boxed region in top image, at a different Z-section. White arrowheads denote RNA signals of LTR retrotransposons in the apical membrane of somatic cells in (F) and in the oocyte in (G’, G”). Somatic membranes are marked by myristoylated GFP. Scale bars (F-H): 10 μm.

LTR retrotransposons are generally confined to the cells in which they are expressed, and in animals their evolutionary survival strategy therefore relies on expression and generation of new genome insertions in the germline. A notable exception is found in the *Gypsy* group of *Metaviridae*, in which some members encode an *env* gene (Fig. 1A). Previous work has demonstrated a strong correlation between a functional *env* gene and exclusive expression in somatic gonadal cells, enabling these elements to infect the germline. In contrast, *Gypsy* LTR retrotransposons lacking an *env* gene are expressed specifically in the germline (Senti et al., 2023).

To identify additional LTR retrotransposons with potential soma-to-germline infectivity, we systematically analyzed their repertoire in *D. melanogaster* using two complementary approaches. First, we inactivated the piRNA pathway specifically in somatic ovarian cells using transgenic RNAi (*vreteno*-RNAi) (Handler et al., 2011), and determined LTR retrotransposon expression using RNA-seq of ovaries. As expected, *Gypsy* group elements with functional *env* were derepressed compared to control, while those lacking *env* were not (Fig. 1B) (Senti et al., 2023). However, all six elements from an additional *Metaviridae* group, the MDG1 clade, were also strongly derepressed by 24-fold despite lacking an *env* gene (Kapitonov & Jurka, 2003). Second, we analyzed the silencing spectrum of the piRNA pathway in the ovarian soma by examining the somatic master piRNA source locus, *flamenco* (Brennecke et al., 2007; Pélisson et al., 1994). Specifically, we asked whether insertions of any LTR retrotransposon group are enriched in *flamenco*. This analysis revealed that, in addition to *env*-encoding *Gypsy* group elements, one additional group is significantly enriched in *flamenco*, the abovementioned MDG1 clade (Fig. 1C). Taken together, LTR retrotransposons of the MDG1 group are expressed in somatic follicle cells under permissive conditions, and the host has evolved somatic piRNA defense against them.

To confirm that the somatic expression of MDG1 LTR retrotransposons is an intrinsic trait and not due to insertions affected by somatically expressed genes, we selected the most highly expressed MDG1 retrotransposon in piRNA pathway knockdown conditions, called *412*, for transcriptional reporter analysis. To this end, we cloned the *lacZ* coding sequence downstream of the LTR of *412*, which harbors the promoter and *cis*-regulatory elements required for expression (Johnson, 2019; Yuki et al., 1986). As expected, the reporter was not expressed in control ovaries, consistent with abundant *412* antisense piRNAs (fig. S1B). However, in ovaries with somatic piRNA pathway knockdown, we observed strong X-Gal staining exclusively in somatic follicle cells (Fig. 1D–E). In contrast, the reporter was not active in ovaries with defective germline piRNA pathway, as opposed to a reporter for a known germline-expressed LTR retrotransposon, *Burdock*, used as a positive control (Fig. 1E bottom) (Senti et al., 2023).

Given these observations, we hypothesized that somatically expressed MDG1 group retrotransposons evolved means to invade the germline, despite them lacking an *env* gene or the sequence space for an unknown open reading frame downstream of *pol* (fig. S1A). To test this, we used single molecule Fluorescence In Situ Hybridization (smFISH) to track the RNA genomes of MDG1 group retrotransposons in ovaries with a somatic piRNA pathway knockdown. During early oogenesis, abundant *412* RNA molecules were found exclusively in nuclei and cytoplasm of somatic follicle cells (Fig. 1F, fig. S1C). However, from stage 6–7 onward, *412* transcripts accumulated at the apical membrane of somatic follicle cells, close to the oocyte, and were also detected inside the oocyte in discrete foci despite an intact germline piRNA pathway (Fig. 1G). The presence of *412* transcripts in the oocyte was surprising, given the strict separation between germline and soma compartments with no cytoplasmic continuity in *Drosophila*. In control ovaries with intact piRNA/PIWI pathway, *412* RNAs were not detectable in somatic cells nor in the oocyte (Fig. 1H). Similar results were observed for the related elements *Stalker2* (Fig. 1F–H) and *Mdg1* (fig. S1D–F), suggesting that MDG1 group retrotransposons are capable of efficient soma-to-germline transmission.

Collectively, our findings indicate that *env*-less LTR retrotransposons of the MDG1 group have adapted viral traits, including somatic expression and a cell-cell transmission strategy, to target the oocyte genome from adjacent somatic cells, thus mimicking infectious retroviruses.

### The LTR retrotransposons 412 and Stalker2 exhibit autonomous infectivity

**Figure 2:**
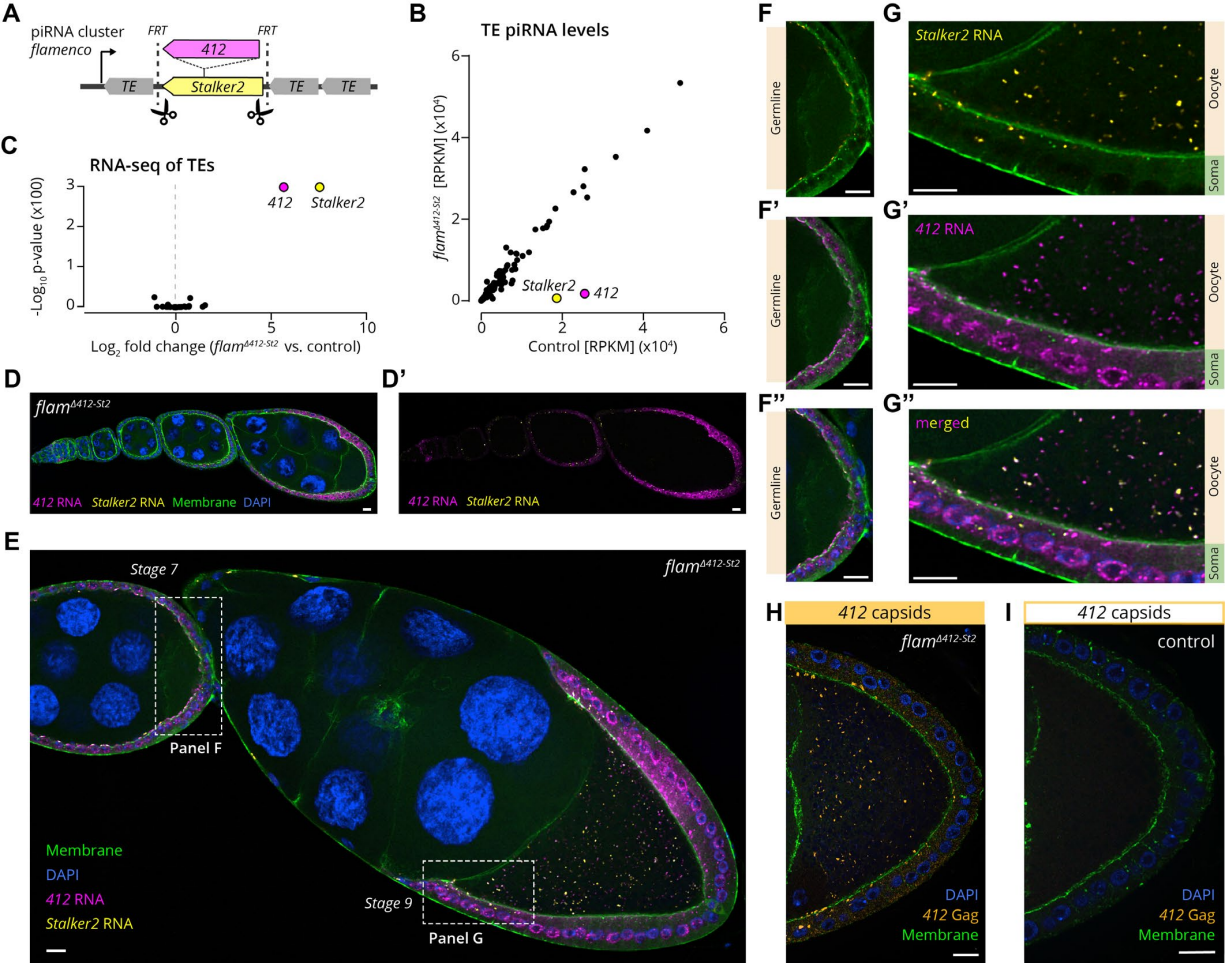
*412* and *Stalker2* retrotransposons autonomously infect the oocyte. **(A)** Schematic organization of the *flamenco* piRNA locus. The *flam*^*Δ412-St2*^ allele was generated by removing a 15.5 kb region encompassing the *412-Stalker2* nested insertion. **(B)** Small RNA sequencing of piRNAs from *flam*^*Δ412-St*^ ovaries, compared to control ovaries (identical genetic background). Each datapoint represents the number of antisense reads mapped to a TE consensus sequence, normalized as reads per kilobase of TE per million mapped microRNAs (RPKM). **(C)** Volcano plot of whole transcriptome poly(A)-enriched RNA-seq, from *flam*^*Δ412-St2*^ ovaries, compared to control ovaries (n=3). Each datapoint represents the number of reads mapped to a TE consensus sequence. **(D-G)** Dual smFISH of *flam*^*Δ412-St2*^ ovaries for *412* RNA (magenta) and *Stalker2* RNA (yellow) in **(D)** early developmental stages (1–7) **(E)** later developmental stages (7 and 9). **(F-G)** Zoomed-in regions from (E), showing absence of oocyte-localized signal for *412* and *Stalker2* in stage 7, whereas in (G) stage 9 there is smFISH signal for both *412* and *Stalker2* within the oocyte. **(H)** Whole-mount immunofluorescence of *flam*^*Δ412-St*^ ovaries, showing somatic apical accumulation and oocyte localization of 412 Gag proteins. **(I)** Same as (H), but for control ovaries (identical genetic background). For (D-I), GFP-labelled myosin II (green) is used to demarcate both the oocyte and soma membranes. Scale bar: (D-I) 10 μm.

Retroviruses infect cells through their Envelope protein, a fusogenic transmembrane glycoprotein that binds to cell surface receptors and facilitates entry via viral-cell membrane fusion. We reasoned that the cell-to-cell transmission observed for MDG1 group retrotransposons might depend on complementation or pseudotyping by Envelope proteins of *Gypsy* group retroviruses, which are also expressed in piRNA deficient follicle cells (Barckmann et al., 2018; Teysset et al., 1998). To test this, we generated flies that are permissive exclusively for MDG1 group retrotransposons. In *D. melanogaster*, almost all transposon-silencing piRNAs in somatic cells originate from the master control locus *flamenco* (Brennecke et al., 2007; Malone et al., 2009). In the reference genome strain, *iso-1*, nested antisense insertions of the MDG1 group elements *412* and *Stalker2* are located ~8kb downstream of the *flamenco* transcriptional start site, flanked by genome-unique sequence stretches (Zanni et al., 2013). Using CRISPR genome editing of the *iso-1* X-chromosome, we inserted two FRT sites flanking the *412*-*Stalker2* locus and excised the intervening 15.5kb sequence with the FLP recombinase (Fig. 2A). To validate the resulting *flamenco*^Δ*412*-*St2*^ allele, we sequenced ovarian piRNAs from two independently generated homozygous mutant lines. The targeted *flamenco* deletion did not change the piRNA pools targeting any transposon, except for *412* and *Stalker2*, whose antisense piRNA levels were reduced ~15 and ~30-fold, respectively (Fig. 2B; fig. S2A). At the same time, differential gene expression analysis based on poly(A)-enriched RNA-seq confirmed that *412* and *Stalker2* were the only de-repressed transposons with significantly increased sense transcript levels (~50-fold; Fig. 2C; fig. S2B–C). The *flam*^*Δ412-St2*^ allele is therefore an ideal tool to study MDG1 group retrotransposons without confounding impacts from *env*-encoding *Gypsy* group retroviruses.

To investigate whether *412* and *Stalker2* are capable of autonomous cell-to-cell transmission, we tracked their RNA genome in *flam*^*Δ412-St2*^ mutant ovaries using smFISH. As in the somatic piRNA pathway knockdown background, *412* and *Stalker2* transcripts were strongly expressed (Fig. 2D–E). During early oogenesis stages, transcripts of both elements showed distinct accumulations at the apical membrane of follicle cells (Fig. 2D–F). By stage 9, smFISH foci were also detected within the oocyte (Fig. 2E, G). We did not observe any smFISH signal in the cytoplasm of nurse cells, which are germline cells connected to the oocyte via ring canals (Fig. 2E). These findings suggest a specific cue or cellular organization that restricts *412* and *Stalker2* transfer exclusively to the oocyte, beginning mid-oogenesis.

To further test whether entire retroviral particles are transmitted, we analyzed the spatio-temporal distribution of potential 412 capsids using a specific polyclonal antibody targeting the CA (capsid) region of the Gag protein. Gag immunofluorescence signals were detected at the follicle cell membranes and within the oocyte, paralleling the RNA localization (Fig. 2H, I), which strongly suggests that entire *412* virus-like particles, encompassing the RNA genome inside a capsid, are capable of cell-to-cell transmission.

Altogether, the *412* and *Stalker2* retrotransposons are autonomous in their ability to transmit both their RNA genome as well as their capsid from somatic follicle cells into the oocyte and must employ a mechanism distinct from that of enveloped retroviruses.

### Two accessory small ORFs are expressed by MDG1 group retrotransposons

**Figure 3:**
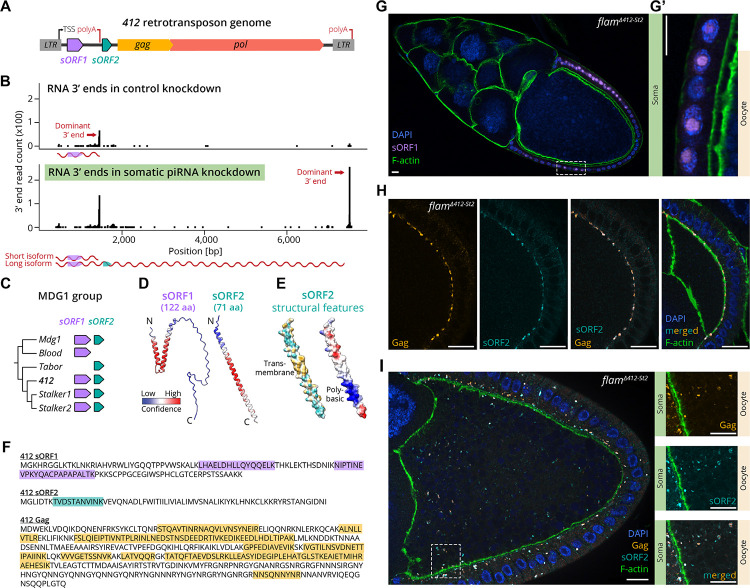
Characterization of the two unique sORFs, sORF1 and sORF2, encoded in *412* genome. **(A)** Schematic of the genomic features encoded by MDG1 retrotransposons, exemplified by the *412* consensus sequence. Features include flanking LTRs, capsid-encoding *gag* and enzymatic machinery-encoding *pol*. Two short ORFs (*sORF1* and *sORF2*), with a predicted premature transcription termination site (polyA) between them, are located upstream of *gag*. TSS, transcriptional start site. **(B)** Mapping of 3’ ends of long-read direct RNA reads corresponding to the *412* consensus sequence, in control ovaries (*Tj*>*Gal4, arr2-RNAi*, top) and somatic piRNA pathway knockdown ovaries (*Tj*>*Gal4, vret-RNAi*, bottom). The Y-axis indicates the number of reads, and the illustration represents the short and long isoforms produced based on the polyA site that is predominantly used. **(C)** Phylogenetic tree of the MDG1 clade based on *pol* alignment, with presence or absence of intact *sORF1* and *sORF2* sequences within the TE consensus sequences of *D. melanogaster* reference genome *iso-1*. Branch lengths are not to scale. **(D)** AlphaFold3 structural predictions for 412 sORF1 and sORF2, with cartoon representation color-coded by pLDDT confidence score (red: high, blue: low). **(E)** Notable features in sORF2 AlphaFold3 predicted structure: left, surface hydrophobicity (cyan: low, yellow: high); right, electrostatic surface potential (red: acidic, blue: basic). **(F)** Mass-spectrometry detection of peptides corresponding to sORF1 (purple), sORF2 (cyan) and Gag (orange) sequences in somatic piRNA pathway knockdown ovaries and OSCs. **(G)** Immunofluorescence staining of a stage 10 *flam*^*Δ412-St2*^ follicle with α-sORF1 antibody (purple). (G’) Zoom-in of somatic nuclei in the boxed region in (G). **(H)** Co-localization of α-sORF2-N (cyan) and α-Gag (orange) on the apical somatic cell membranes in an early stage follicle. **(I)** Immunofluorescence signals of α-sORF2-C (cyan) and α-Gag (orange) often co-localize within the oocyte in later stage follicles. Right panels show zoomed-in region from I at a different Z-plane. For (G-I) F-actin is labelled with phalloidin (green) to demarcate cortical actin near the plasma membrane. Scale bars (G-I): 10 μm.

We hypothesized that members of the MDG1 group use an alternative infectivity factor to mediate cell-to-cell transmission. Two small open reading frames (ORFs) upstream of *gag*, denoted *sORF1* and *sORF2*, have been identified as putative accessory ORFs in the MDG1 group (Fig. 3A) (Avedisov et al., 1990; Costas et al., 2001; Makarova, 1997; Yuki et al., 1986). While their low non-synonymous/synonymous mutation rates suggest functionality (Costas et al., 2001; Mugnier et al., 2005), it remains unclear whether these short predicted ORFs (~110 amino acids for sORF1 and ~70 amino acids for sORF2) are indeed transcribed and translated into functional proteins and what their physiological roles might be.

To this end, we performed long-read direct RNA sequencing on ovaries and a cultured cell line derived from ovarian somatic cells (OSCs), where in control conditions *Gypsy* retroviruses and MDG1 group retrotransposons are similarly repressed by the piRNA pathway. Upon somatic piRNA pathway knockdown, we detected continuous ~7 kb transcripts of *412*, *Mdg1*, *Tabor*, *Stalker2* and *Blood* that span LTR to LTR, showing no evidence of splicing, and include the *sORF1* and *sORF2* region (Fig. 3B and fig. S3–4). Interestingly, an additional short ~1.5 kb transcript terminating immediately after *sORF1* was also expressed, consistent with a previously predicted alternative polyadenylation site between *sORF1* and *sORF2* (Cherkassova et al., 1991). This short transcript could be detected at low levels for most MDG1 elements also in the control ovaries and OSCs. Our data therefore suggest that in wildtype conditions, a short *sORF1* transcript is expressed, while full-length transcripts spanning both *sORFs* together with *gag* and *pol* are repressed by the piRNA pathway.

Based on Berkeley *Drosophila* Genome Project (BDGP) and Repbase TE consensus sequences, *sORF1* and *sORF2* genes are encoded in all members of the *D. melanogaster* MDG1 group, except for *Tabor* and *Blood*, which harbor deletions disrupting *sORF1* and *sORF2*, respectively (Fig. 3C) (Bao et al., 2015; Costas et al., 2001; Kaminker et al., 2002). Protein structure predictions using AlphaFold3 (Abramson et al., 2024) revealed a potential helix-turn-helix motif for sORF1, while sORF2 likely forms an alpha-helix with a central hydrophobic domain followed by an adjacent polybasic region (Fig. 3D–E). To determine whether the two sORFs are translated, we conducted untargeted proteomics on somatic piRNA-deficient ovaries and OSCs. We identified high confidence peptides corresponding to sORF1 for *412*, *Mdg1*, *Blood* and *Stalker2* and to sORF2 for *412*, *Mdg1* and *Stalker1* (Fig. 3F, fig. S5. We note that the detection of sORF2 is challenging due to the limited number of suitable tryptic peptides).

To gain insight into their potential functions, we determined the cellular localization of the *412* sORF1 and sORF2 proteins in ovaries using polyclonal antibodies. Antibody specificity was validated by the strongly elevated signal in *flam*^*Δ412-St2*^ ovaries versus control ovaries from flies that were genetically identical except for the targeted *flamenco* deletion (fig. S6A–D). Whole mount immunofluorescence experiments revealed that sORF1 is exclusively expressed in somatic follicle cells, where it localizes to a subnuclear compartment (Fig. 3G). No signal was detected in the oocyte, even at later developmental stages when *412* transmission occurs. We conclude that sORF1 likely functions early in the transposon life cycle and is not directly involved in the transmission process.

In contrast, two independent antibodies targeting the N or C-terminus of *412* sORF2 revealed a plasma membrane-corresponding staining in follicle cells (Fig. 3H and fig. S6B, E). We obtained similar results in flies expressing a C-terminally HA-tagged sORF2 transgene in the soma, where the HA antibody and the sORF2 N-terminal antibody yielded overlapping signals (fig. S6C). During previtellogenic stages (up to stage 6), sORF2 localized to the apical side of follicle cells, which faces the germline (fig. S6E). Notably, these membranal sORF2 accumulations were also enriched with viral capsids, as indicated by co-localization with the Gag-targeting antibody and with cortical actin marker phalloidin (Fig. 3H). At later developmental stages (from stage 7 onwards), we detected abundant sORF2 signal also within the oocyte, often overlapping with Gag, in accordance with the timing of *412* transmission into the oocyte observed by smFISH (Fig. 3I).

In sum, the plasma membrane localization of sORF2 and its close association with viral capsids suggest that sORF2 may play a role in facilitating the cell-to-cell transmission of MDG1 group retrotransposons.

### sORF2 is a transmembrane protein with hallmarks of cell-cell fusogens

**Figure 4:**
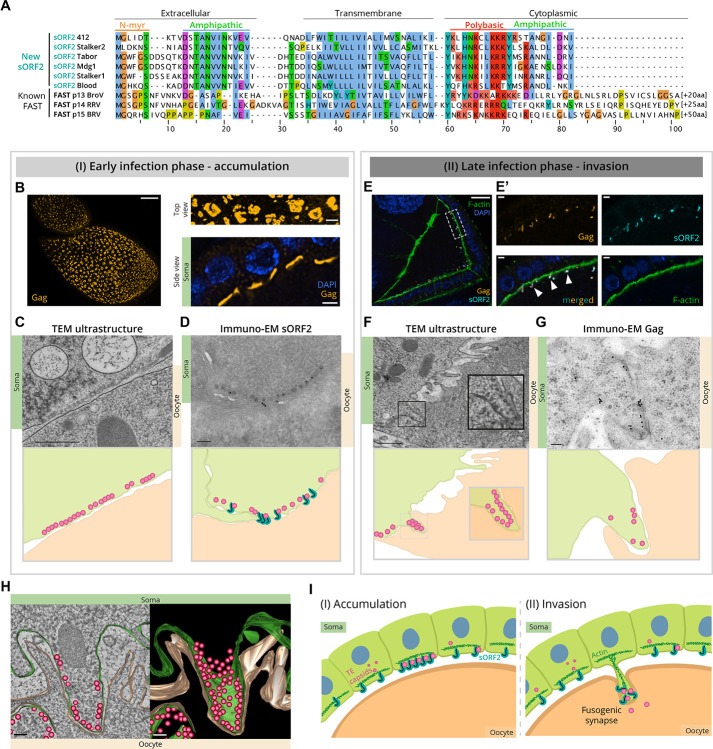
sORF2 proteins resemble FAST proteins, and promote cell fusion by facilitating proximity of membranes. **(A)** Multiple sequence alignment of sORF2 proteins from *D. melanogaster* and three previously described FAST proteins. p13 from bat Broome reovirus (BroV, ACU68609.1); p14 from reptilian orthoreovirus (RRV, AAP03134.1); p15 from baboon orthoreovirus (BRV, AAL01373.1). sORF2 proteins of *Stalker1* and *Stalker2* lack the predicted canonical N-myristoylation motif (N-myr, MGxxxS/T) but share all other features. *Blood* consensus sequence lacks an intact sORF2, but several heterochromatic insertions supported a *Blood* variant with the presented sORF2 sequence. Clustal X coloring scheme labels conserved residues according to amino acid profile, additional Arg and Lys residues in the polybasic region are shown in light red. **(B)** Left, Immunofluorescence Z-stack projection of stage 7 *flam*^*Δ412-St2*^ follicles expressing GFP-labelled myosin II (green)stained with α-Gag (orange) showing accumulations of capsids within the somatic follicle cell layer covering the oocyte. Scale bar: 10 μm. Top right, zoom-in of the capsid accumulation pattern. Scale bar: 2 μm. Bottom right, mid-section of the follicle showing 412 capsid accumulations along the somatic apical membranes. Scale bar: 2 μm. **(For C-D, F-G)** Upper panels, Transmission (TEM) and immuno-EM images. Lower panels, schematic representation of EM images, somatic cells (green) with observed capsids (pink) are in close proximity to the oocyte membrane (beige) and sORF2 proteins (cyan). **(C)** TEM of capsid accumulations along somatic apical membranes in stage 7 *flam*^*Δ412-St2*^ follicle. Scale bar: 500 nm. **(D)** Immuno-EM of *flam*^*Δ412-St2*^ stage 7 follicle using α-sORF2-C antibodies, labelled with secondary antibody-conjugated gold particles (black dots in upper panel, cyan in lower schematic), revealing sORF2 localization near accumulated capsids. Scale bar: 100 nm. **(E)** Immunofluorescence of *flam*^*Δ412-St2*^ ovaries co-stained with α-Gag (orange), α-sORF2-C (cyan) and phalloidin (green), showing co-localization in protrusions extending into the oocyte (white arrowheads). Scale bars: 10 μm, zoom-in panels on right: 2 μm. **(F)** TEM of an invasive protrusion filled with capsids in *flam*^*Δ412-St2*^ follicle, extending into the oocyte. Adjacent physiologically normal spacing between membranes and typical microvilli are shown for comparison. Scale bar: 500 nm. Inset, zoom-in of protrusion with indistinguishable soma and oocyte membrane. **(G)** Immuno-EM of *flam*^*Δ412-St2*^ ovaries using α-Gag antibody, with gold particles (black dots) corresponding to 412 capsids within an invading protrusion. Scale bar: 100 nm. **(H)** Snapshot of electron tomography segmentation and 3D rendering of an invasive protrusion in *flam*^*Δ412-St2*^ ovaries, filled with 412 and Stalker2 viral-like particles (pink). The somatic membrane (green) is closely associated with the oocyte membrane (beige), with minimal separation between them. Scale bar: 100 nm. **(I)** Proposed model for soma-to-oocyte infectivity of MDG1 LTR retrotransposons. (Left) somatically expressed transmembrane sORF2 proteins (cyan) are closely associated with LTR retrotransposon capsids (pink). Upon local clustering and additional unknown cues, cortical actin-remodeling (green) is initiated (right), promoting the formation of invasive protrusions toward the oocyte membrane. Once the membranes are close enough in a fusogenic synapse structure, transient fusion between somatic and oocyte membranes enables LTR retrotransposons to enter the oocyte.

To gain insights into sORF2 function, we examined conserved features in MDG1 group sORF2 proteins using a multiple sequence alignment (Fig. 4A). This analysis revealed a highly hydrophobic central domain predicted to function as transmembrane domain with high-confidence, flanked by an N-terminal ectodomain and a C-terminal cytoplasmic tail (see Materials and Methods). Furthermore, all sORF2s harbor an amphipathic motif in their ectodomain and a positively charged basic patch followed by an amphipathic motif in their cytoplasmic tail (fig. S7). Finally, four out of six sORF2 proteins harbor a predicted N-terminal glycine myristoylation motif (MGxxxS/T), a fatty acid modification known to promote protein-membrane association and localization to specific membrane microdomains, especially in the context of viral proteins (Wang et al., 2021).

Collectively, these structural features are highly reminiscent of fusion-associated small transmembrane (FAST) proteins, which mediate cell-cell fusion rather than classical viral-cell fusion (Duncan, 2019). FAST proteins have been mainly identified in several fusogenic reoviruses and rotaviruses (order *Reovirales*), which are non-enveloped, double-stranded RNA viruses that infect a wide range of hosts (Ciechonska & Duncan, 2014; Diller et al., 2019). Recent studies have shown that FAST proteins function as cell-cell fusogens by multimerizing in specific membrane domains and recruiting the actin cytoskeleton to generate localized mechanical force, pushing two cellular membranes together and disrupting them to drive membrane fusion (Chan et al., 2020, 2021). Since FAST proteins are short (~100–200 residues) and exhibit highly divergent sequences, primary sequence-based homology search cannot be used to establish a link between sORF2 and FAST proteins. Nevertheless, the MDG1 group sORF2 and reoviral FAST proteins share a striking resemblance of domain organization and multiple predicted motifs (Fig. 4A).

### 412 sORF2 and capsids localize to fusogenic synapses between follicle cell protrusions and the oocyte membrane

In the *Drosophila* egg chamber, somatic follicle cells form apical microvilli - actin-filled membranous extensions characteristic of epithelial cells - that project toward the oocyte (Schlichting et al., 2006). We wondered whether MDG1 group sORF2 proteins function in an analogous manner to reoviral FAST proteins, by forcing somatic microvilli membranes into close contact with the oocyte membrane to enable local cell-cell fusion and capsid transmission. To test this hypothesis, we performed extensive light and electron microscopy (EM) studies, leveraging the natural contrast provided by viral capsids, to examine the follicle cell-oocyte interface in *flam*^*Δ412-St2*^ and control ovaries.

Using EM, we observed that the soma-to-oocyte infection process occurs in two distinct phases: In the first phase, 412 virus-like particles and sORF2 proteins accumulate in discrete clusters along the apical membranes of somatic follicle cells, in agreement with our immunofluorescence findings (Fig. 3H, 4B–C). Transmission EM ultrastructure analysis revealed that the abundant capsid structures measured 42+/−5 nm in diameter and exhibited a characteristic dark outer rim and a lighter inner region, consistent with the size and pattern reported previously for *Gypsy* retroviruses (Lécher et al., 1997). Cryo-immunoEM experiments with antibodies targeting 412 Gag and sORF2 confirmed that many of the observed virus-like particles correspond to 412 capsids and closely associated sORF2 proteins. However, given the co-expression of *Stalker2*, we anticipate the virus-like particles to represent a mix of 412 and Stalker2 capsids (Fig. 4D, fig. S8).

The second phase is characterized by the formation of invasive protrusions at the apical membrane of follicle cells which extend into the oocyte (Fig. 4E–H, fig. S8C). These cellular protrusions were exclusively observed within the ultrastructure of *flam*^*Δ412-St2*^ ovaries and typically had enlarged bulges at their tips, each filled with dozens of capsids (Fig. 4F–G). Unlike regular microvilli, which are sleek, slender and devoid of capsids, these specialized structures were distinguishable by their bulging morphology and tight encapsulation by the oocyte membrane (fig. S8F–G). EM tomography experiments further revealed that capsids are dispersed along the periphery of the invasive protrusion bulges, reinforcing their role as functional structures for viral transmission (Fig. 4H and Supplementary Movie S1).

In stage 6–7 *flam*^*Δ412-St2*^ follicles, the invasive protrusions displayed considerable variation in size, shape, position, and number (fig. S8F–G, S9A–B), likely reflecting different maturation stages in the soma-to-oocyte infection process. Interestingly, these protrusions were rarely observed at earlier (stage 4–5) or later (stage 9–10) developmental stages, suggesting a narrow window of opportunity for soma-to-oocyte transfer. This window corresponds to the time from the onset of microvilli formation to the stage when the secreted vitelline bodies coalesce to form the impenetrable vitelline shell around the oocyte (fig. S9C–D) (King & Koch, 1963; Mallart et al., 2024). We never observed the characteristic protrusions in somatic cells neighboring the nurse cells, although capsids readily accumulated along the apical membrane, corresponding to the first phase only (Fig. 4B, fig. S9E–F). These findings imply the critical involvement of unidentified tissue-specific host factors in the formation of the invasive protrusions.

In order to retain the native ultrastructure of membranes, we used high pressure freezing for the EM ultrastructure experiments, allowing the clear visualization of the lipid bilayers of somatic follicle cell and oocyte membranes. Intriguingly, in several capsid-filled protrusions, we observed that the somatic and the oocyte membranes were indistinguishable, suggesting sites of confined, intimate membrane-membrane interaction that overcame the repulsive forces acting between membranes (Chernomordik & Kozlov, 2003) (notice spacing between membranes in Fig. 4F,H and in fig. S8,9). We propose that these sites represent restricted zones of transient cell-cell fusion, facilitating the transmission of viral-like particles into the large oocyte volume (Fig. 4I).

Based on our detailed cell-biological studies, we suggest that the invasive protrusions function as fusogenic synapses, specialized structures that mediate localized membrane proximity and fusion between adjacent cells (J. H. Kim & Chen, 2019; Shilagardi et al., 2013). These findings strongly support a model in which sORF2 drives the formation of these protrusions, similar to FAST proteins, enabling MDG1 group retrotransposons to transmit directly from somatic follicle cells into the oocyte.

### sORF2-encoding LTR retrotransposons are widespread in insect genomes

**Figure 5:**
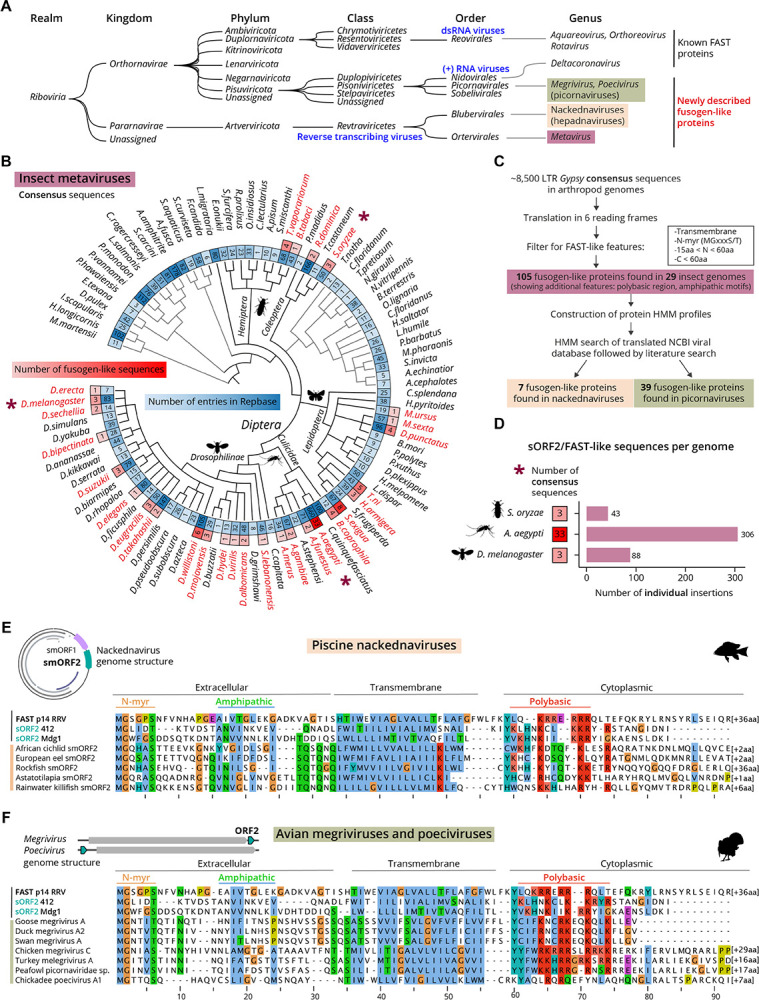
sORF2/FAST-like sequences are abundant and widespread among non-enveloped viruses. **(A)** Taxonomic tree of all viruses and TEs described in this study. **(B)** Taxonomic tree of arthropod genomes annotated in Repbase for *Metaviridae* consensus sequences. Inner circle (blue shades) – number of TE entries in the database per species. Outer circle (red shades) – number of *sORF2/FAST*-like consensus sequences found per species. Insect genomes analyzed in (D) are labelled with asterisk. **(C)** Schematic representation of computational pipeline to identify sORF2/FAST-like proteins in additional genomes. **(D)** Number of individual sORF2/FAST-like sequences in LTR retrotransposon insertions of three insect genomes. **(E-F)** Multiple sequence alignment of a subset of sORF2/FAST-like (E) smORF2 proteins found in fish-infecting nackednaviruses (F) ORF2 proteins from bird-infecting picornaviruses, and their typical genome organization. Clustal X coloring scheme labels conserved residues according to amino acid profile, additional Arg and Lys in the polybasic region are shown in light red.

Many organisms share the general architecture of ovarian follicles, where supportive somatic cells and their membrane extensions closely interact with the oocyte (Clarke, 2022; Jaglarz et al., 2008). To explore whether the sORF2-mediated transfer demonstrated in *Drosophila* represents a broader phenomenon, we examined the prevalence of sORF2/FAST-like proteins in LTR retrotransposons (Fig. 5A). We first performed a non-exhaustive search on ~8,500 representative *Metaviridae* consensus sequences in arthropods taken from the Repbase database (Bao et al., 2015). Due to the short length of sORF2 proteins, direct sequence-based homology searches were unproductive. Instead, we translated all possible ORFs from the consensus sequences and filtered for short ORFs harboring a predicted transmembrane domain and N-myristoylation motif (Fig. 5B–C, see Materials and Methods). This approach identified 105 high-confidence hits across 29 organisms, ranging from flies and mosquitoes (*Diptera*), through butterflies and moths (*Lepidoptera*) to beetles (*Coleoptera*), (Fig. 5B). The highest diversity of sORF2/FAST-like sequences within a single organism, 33, was found in the mosquito *Aedes aegypti*, the primary vector of human-pathogenic viruses including dengue, Zika, and yellow fever viruses, which has an exceptionally large, repeat-rich genome (Daron et al., 2024). All *sORF2/FAST*-like candidates were found in the forward orientation relative to the *gag* and *pol* ORFs, suggesting they depend on the primary transcriptional start site and do not utilize an alternative promoter (fig. S10A). In some LTR retrotransposons of *Lepidoptera*, we have identified cases where *sORF2* genes are encoded downstream of *pol* instead of upstream of *gag* (7/22 consensus sequences in *Lepidoptera*).

A closer examination of the multiple sequence alignment of the 105 high-confidence sORF2/FAST-like sequences revealed additional conserved features previously described for FAST proteins, including a juxtamembrane polybasic region and amphipathic motifs (fig. S7C), despite these not being part of the initial search criteria. sORF2/FAST-like sequences ranged in length from 63 to 107 amino acids, with shorter variants usually found in flies and mosquitoes, and longer ones in butterflies, moths, and beetles. By comparison, known FAST proteins in *Reoviridae* range from 80 to 200 residues (Duncan, 2019; Veletanlic et al., 2023). Interestingly, in both known FAST proteins and newly found sORF2-like proteins, the primary variation in overall length arises from differences in the endodomain, while the ectodomain is rather constant at around 30 residues (fig. S10B). This conserved ectodomain length may reflect a functional role in membrane biology and could serve as a useful narrowing filter for identifying additional *bona fide* sORF2/FAST-like proteins. Finally, a previously undescribed feature that has emerged with the increased number of available sequences is a conserved Tyr residue immediately downstream of the transmembrane domain. This residue is often found at the interfacial boundary of transmembrane proteins, forming part of the “aromatic belt” (Baker et al., 2017) (fig. S10B).

Estimating the prevalence of *sORF2/FAST*-like genes within a genome is challenging because consensus sequences fail to capture the full extent of heterogeneous multi-copy TE insertions. To overcome this limitation, we analyzed three high-quality TE-curated insect genomes, systematically scanning them for sORF2/FAST-like sequences within LTR retrotransposon insertions. Using this approach, we identified 88 insertions in the *Drosophila melanogaster iso-*1 reference genome, mostly belonging to *412*, *Mdg1*, *Tabor* and *Blood* retrotransposons (Fig. 5D). In the mosquito *A. aegypti*, 306 LTR retrotransposon insertions with *sORF2/FAST*-like sequences, of which 296 corresponded to the 33 representative consensus sequences, were found. Lastly, in the major agricultural pest *Sitophilus oryzae* (rice weevil), 43 *sORF2/FAST*-like sequences were found in the context of LTR retrotransposon insertions. Altogether, considering the enormous diversity of insect species and the abundance of sORF2/FAST-encoding LTR retrotransposons within them, our findings highlight a highly successful viral-like evolutionary strategy of LTR retrotransposons to prevail within their hosts. Further supporting this notion is the evidence that the *412* retrotransposon invaded *D. melanogaster* genome less than 200 years ago, and has since successfully spread to every sampled natural population of *D. melanogaster* (Scarpa et al., 2024).

### sORF2/FAST-like proteins suggest an Envelope-independent transmission strategy also in hepadnaviruses and picornaviruses

If FAST and sORF2-like proteins enable cell-to-cell transmission in non-enveloped fusogenic reoviruses and LTR retrotransposons, respectively, we hypothesized that other non-enveloped viruses might employ similar strategies. Building on the large repertoire of sORF2/FAST-like proteins identified in insect genomes, we constructed hidden Markov model (HMM) profiles of sORF2s and used them to scan the NCBI viral database. This approach revealed putative sORF2/FAST-like proteins in two additional, entirely unrelated viral families: *Hepadnaviridae* and *Picornaviridae*.

Hepadnaviruses are considered distant relatives of retroviruses (Fig. 5A) (Magnius et al., 2020). They also undergo reverse transcription as part of their life cycle and encode for an Envelope protein, although distinct from that of retroviruses. The most extensively studied member of this group is human hepatitis B virus, whose phylogenetic origin remains unresolved. In an effort to elucidate the time and source of *envelope* acquisition, Lauber *et al*. described a family of non-enveloped hepadnaviruses in teleost fish, which they named “nackednaviruses” (Lauber et al., 2017). These fish viruses exhibit many canonical structural and functional features of hepatitis B virus, except for the *envelope* gene, leaving the mechanism of how these viruses enter cells unclear.

Interestingly, the authors note that all thirteen described nackednaviruses encode two small ORFs, designated “*smORF1*” and “*smORF2*”, positioned in the viral genome in the same orientation and upstream of the capsid-encoding core gene (*orf C*). We propose *smORF2* encodes for a sORF2/FAST-like protein, as all eleven complete smORF2 proteins are 75–114 amino acids long, contain a predicted transmembrane domain with N-outside/C-inside topology, and exhibit amphipathic motifs in both the extracellular and cytoplasmic domains. Additionally, they feature a polybasic region just C-terminal of the membrane domain, and seven encode a canonical N-myristoylation motif (Fig. 5E, fig. S11A). Available nackednaviral RNA-derived sequences indicate that both *smORFs* are transcribed and not spliced out. Based on a later study of the nackednavirus capsid, no structural change of the capsid occurs upon pH change, in contrast to capsids of known non-enveloped viruses, which would argue against a typical endocytic-entry path (Pfister et al., 2023). We thus speculate that nackednaviruses employ a cell-to-cell transmission mode involving their sORF2/FAST-like smORF2 proteins. Direct experimental studies are required to determine the viral and cellular determinants of smORF2 activity.

The second group that was found using the sensitive HMM search was avian picornaviruses, specifically megriviruses and poeciviruses. Unlike most picornaviruses, which encode a single polyprotein, some megriviruses encode an accessory protein, ORF2, of unknown function (Boros et al., 2014). We found many of the characteristic features of FAST proteins as described above in 34 megriviruses sequences, at a conserved position downstream to the main ORF, spanning 67–115 amino acids in length. (Fig. 5F, fig. S11B). Interestingly, pathological reports of turkey hepatitis cases associated with megrivirus infections describe syncytia-like cells in the liver, which the authors note is peculiar since there is no established relationship between syncytia and a picornavirus (Hauck et al., 2014). We suggest the syncytia were formed by the activity of sORF2/FAST-like ORF2 proteins encoded in the megrivirus genome, which is circumstantially lacking from other non-syncytia forming picornaviruses. Similarly, the related poecivirus, the suspected causative agent of the devastating avian keratin disorder in birds, also encodes an unannotated gene for a sORF2/FAST-like protein, however it is positioned upstream of the main ORF (Fig. 5F, fig. S11B) (Zylberberg et al., 2018). Future work is needed to determine what is the evolutionary trajectory and possible shared origin of sORF2/FAST-like proteins found in reoviruses, rotaviruses, LTR retrotransposons, hepadnaviruses, and picornaviruses.

## DISCUSSION

This study provides direct evidence for viral-like infectivity of MDG1 group LTR retrotransposons in *Drosophila*, demonstrated at both the RNA and protein levels. We show that the soma-to-germline transmission occurs independently of Envelope and relies instead on the formation of somatic protrusions that invade the oocyte. In the context of the host germline, MDG1 group retrotransposons are yet another example of breaking the Weismann’s barrier in metazoans, which postulates genetic material is only unidirectionally transferred from germline to soma (Bline et al., 2020; Yoth et al., 2023).

Direct cell-to-cell transmission offers several advantages over Envelope-mediated cell exit and re-entry, such as avoiding extracellular restriction factors and bypassing the need for receptor-mediated internalization. While cell-cell fusion has been described for some retroviruses, such as HIV and the case of conduits formed by HTLV (Bracq et al., 2018; Van Prooyen et al., 2010), these processes are thought to depend on Envelope proteins. Our work demonstrates, for the first time, a functional *env*-independent retrovirus capable of direct transmission.

The sORF2 protein, bearing structural similarity to reoviral FAST proteins—the only known fusogenic proteins encoded by non-enveloped viruses—appears to enable limited and specific cell-cell fusion. Unlike the aggressive fusion seen in fusogenic reoviruses, which leads to extensive syncytium formation, sORF2-mediated fusion appears to be tightly controlled. This regulation likely ensures host fertility while enabling retrotransposon persistence. The mechanism of sORF2-mediated fusion resembles more developmental cell fusogens, such as Myomerger and EFF-1, rather than potent viral fusogens (Sapir et al., 2008). We propose that sORF2 evolved to be temporally and spatially restricted, allowing viral genome transfer only in a subset of cells in a well-defined developmental window. Invasive protruding membrane structures, as seen in fertilization (Satouh & Inoue, 2022), osteoclast fusion (Oikawa et al., 2012), macrophage fusion (Faust et al., 2019), and myoblast fusion (Luo et al., 2022; Shilagardi et al., 2013) are distinctive features of fusion-related processes *in vivo*. However, such structures associated with FAST proteins have not yet been observed in their native tissue context. Our study synthesizes these findings together, suggesting that in the ovary tissue, FAST-like sORF2 proteins either exploit existing actin-rich microvilli or form them *de novo* to bring the somatic and oocyte membranes closely together in a fusogenic synapse structure.

To the best of our knowledge, less than 20 FAST proteins have been reported, mostly within the *Reoviridae* family (Duncan, 2019; Veletanlic et al., 2023) and a few others in coronaviruses (Huang et al., 2016; Sartalamacchia et al., 2024). Our findings reveal that sORF2/FAST-like proteins are encoded also in *Metaviridae*, *Hepadnaviridae* and *Picornaviridae*, substantially expanding the repertoire of known FAST-like proteins across the viral kingdom. Given the multi-copy nature of TEs and the extraordinary diversity of insects, birds, and fish, as well as their short and non-conserved primary amino acids sequence, these proteins are likely far more abundant than previously appreciated. Among these different virus-host interactions, perhaps the most surprising is that of insect LTR retrotransposons, which were thought to be confined to the cells in which they are expressed.

Viruses are known for their ingenious strategies to manipulate host cell biology, often inspiring biotechnological advances. Similar to FAST proteins, which have recently shown promise in gene delivery systems (Brown et al., 2024), sORF2-based mechanisms could inform the development of targeted biotechnological tools or therapeutic strategies. Furthermore, studying host-pathogen interactions in insects may yield valuable insights for controlling mosquito-borne diseases or developing biocontrol agents against agricultural pests. Our work provides fundamental new insights on retrotransposon biology, fusogen evolution, and host-pathogen interactions, with implications for both fundamental and applied sciences.

### Limitations of the study

Despite these advances, our study has some limitations. First, our efforts to recapitulate sORF2-mediated fusion in minimal heterologous experimental systems using standard cell-cell fusion reporter assays have not proven successful so far, likely due to specific requirements for cellular factors and tissue-level architecture that is challenging to replicate in cell culture. Another confounding factor in our study is the encoding of sORF2 as part of the multi-copy transcript of MDG1 group elements, which hinders genetic approaches to determine if sORF2 is indeed required for cell-cell fusion. Lastly, the identification of sORF2/FAST-like proteins remains constrained by our search criteria, which focus on canonical start codons and N-terminal myristoylation motifs, excluding non-canonical or alternative variants, such as palmitoylated proteins seen in some FAST proteins (Duncan, 2019; Shmulevitz et al., 2003). Future studies need to address these limitations to deepen our understanding of sORF2 function and its evolutionary origin.

## Supplementary Material

Supplement 1

Supplement 2

Supplement 3

Supplement 4

Supplement 5

1

## Data Availability

All fly stocks and antibodies generated for this study are available upon request. The sequencing data have been deposited in the NCBI Gene Expression Omnibus (GEO) under the accession number XXX. The mass spectrometry proteomics data have been deposited to the ProteomeXchange Consortium via the PRIDE (Perez-Riverol et al., 2025) partner repository with the dataset identifier PXD061763.
