# Supplementary figures and images for: Direct cell-to-cell transmission of retrotransposons
Source: bioRxiv. 2025 Mar 16:2025.03.14.642691. Preprint. [Version 2] doi: 10.1101/2025.03.14.642691 (PMC11952523; doi:10.1101/2025.03.14.642691)

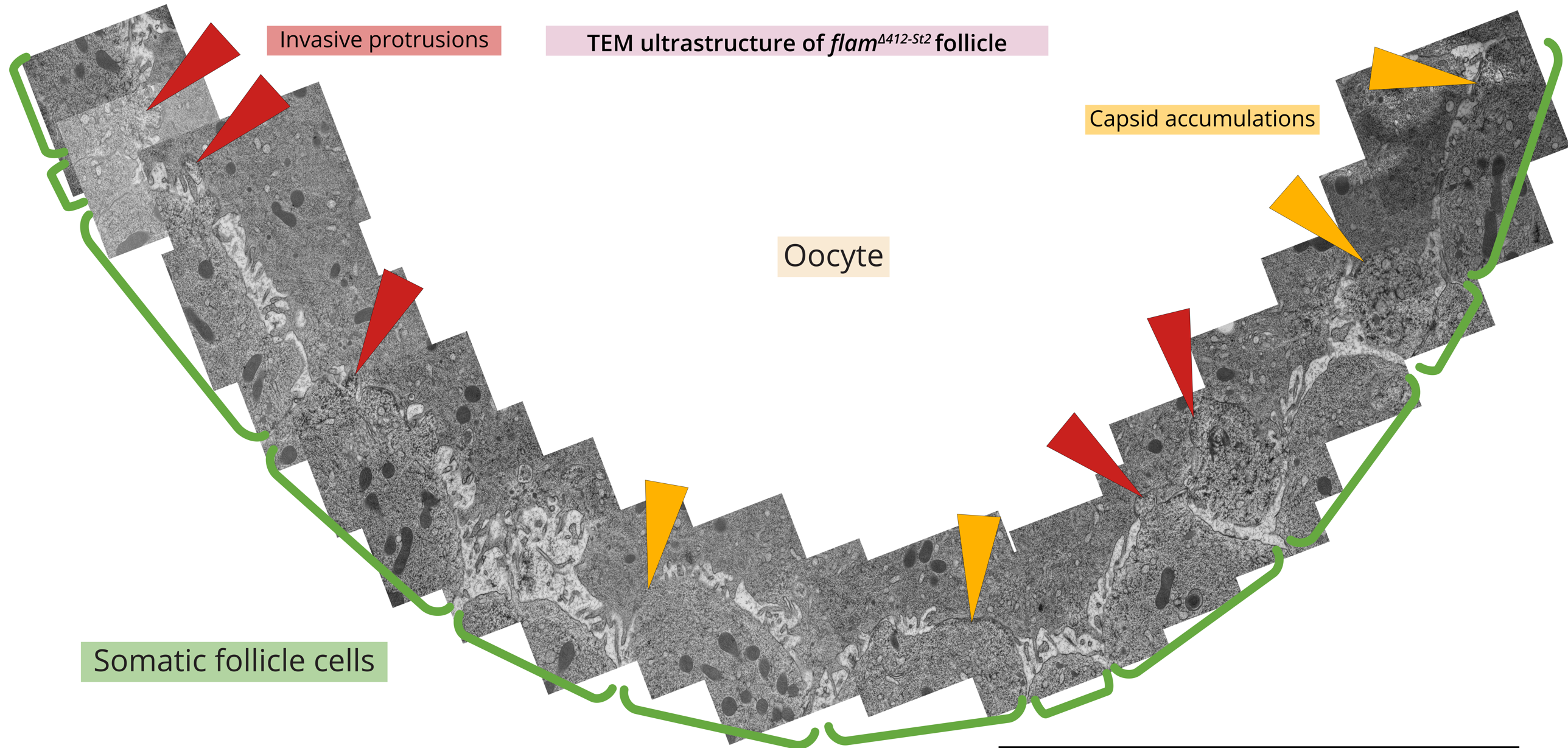

Supplement: Supplement 3 [file media-3.pdf]

TEM ultrastructure of control follicle

Oocyte

Somatic follicle cells

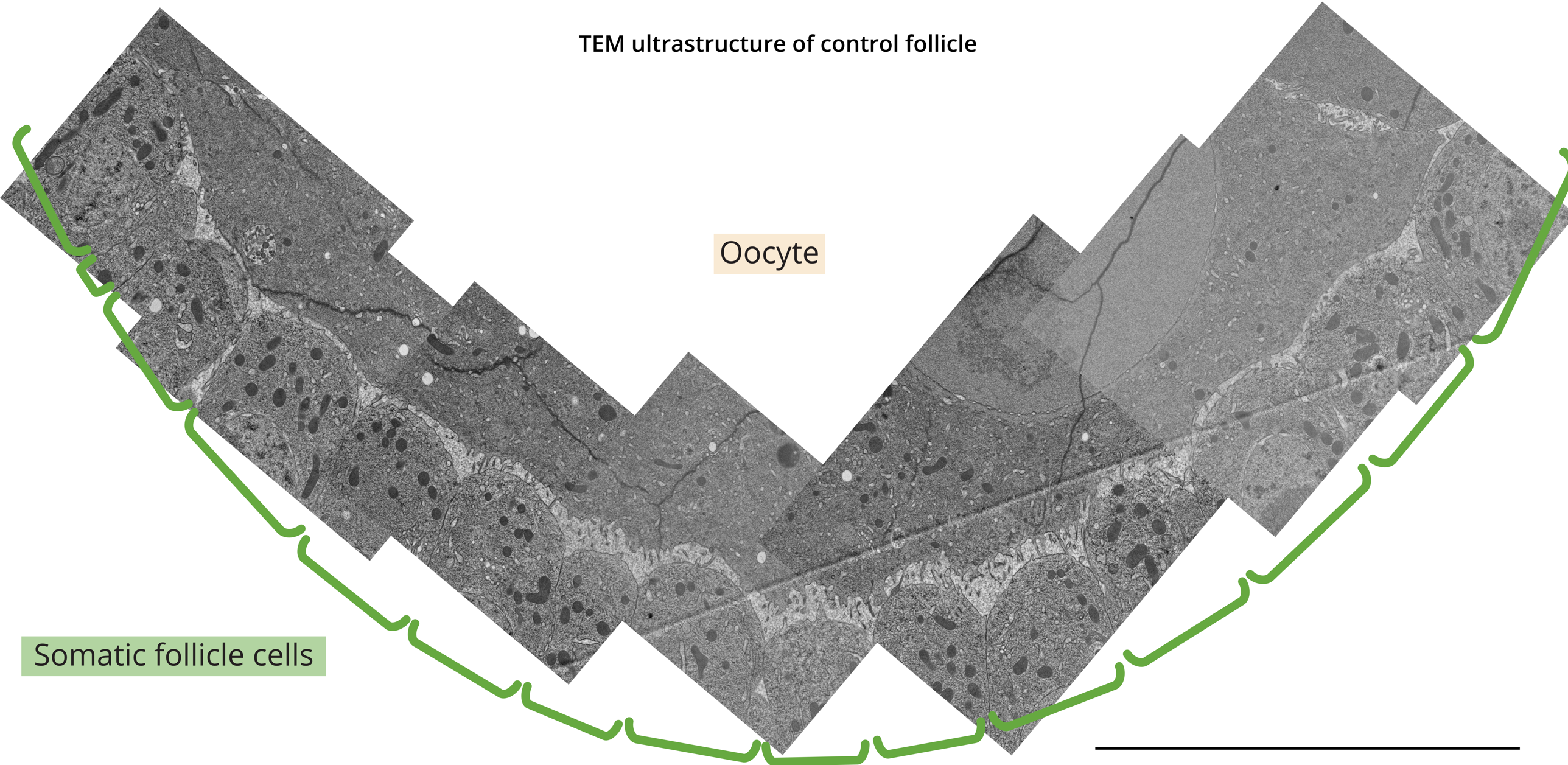

Supplement: Supplement 4 [file media-4.pdf]
