# Supplementary material for: Direct cell-to-cell transmission of retrotransposons
Source: bioRxiv. 2025 Mar 16:2025.03.14.642691. Preprint. [Version 2] doi: 10.1101/2025.03.14.642691 (PMC11952523; doi:10.1101/2025.03.14.642691)
Supplement: 1 [file NIHPP2025.03.14.642691V2-supplement-1.pdf]

bioRxiv preprint doi: https://doi.org/10.1101/2025.03.14.642691; this version posted March 16, 2025. The copyright holder for this preprint (which was not certified by peer review) is the author/funder, who has granted bioRxiv a license to display the preprint in perpetuity. It is made available under aCC-BY 4.0 International license.

Supplementary Materials for

Direct cell-to-cell transmission of retrotransposons

Maya Voichek, Andreas Bernhard, Maria Novatchkova, Dominik Handler, Paul Möseneder, Baptiste Rafanel, Peter Duchek, Kirsten-Andre Senti, Julius Brennecke

Corresponding authors: senti@imba.oeaw.ac.at, julius.brennecke@imba.oeaw.ac.at

The PDF file includes:

Materials and Methods
Figs. S1 to S11

1. **Figure S1:** Robust somatic expression and oocyte transmission of MDG1 retrotransposons
2. **Figure S2:** RNA expression of TEs in *flam*$^{\Delta 412\text{-}St2}$ ovaries
3. **Figure S3:** *412* and *Stalker2* produce long and short transcripts
4. **Figure S4:** Premature transcription termination sites are found in most MDG1 retrotransposons
5. **Figure S5:** Untargeted proteomics evidence for translation of MDG1 retrotransposon ORFs
6. **Figure S6:** Antibodies targeting sORF1 and sORF2 are specific and support distinct cellular localizations
7. **Figure S7:** Transmembrane and amphipathic domains in newly discovered fusogen-like proteins
8. **Figure S8:** Characterization and validation of observed capsids and protrusions in *flam*$^{\Delta 412\text{-}St2}$ follicles
9. **Figure S9:** Invasive protrusions form exclusively in the soma-oocyte interface of *flam*$^{\Delta 412\text{-}St2}$ follicles during a developmental time window
10. **Figure S10:** *sORF2/FAST*-like genes are encoded within *Metaviridae* in insect genomes
11. **Figure S11:** *sORF2/FAST*-like genes are encoded as separate ORFs within non-enveloped viral genomes

Additional supplementary files:

Tables S1 to S2

1. **Table S1:** sORF2/FAST-like sequences identified in this study
2. **Table S2:** Fly genotypes, smFISH probes and siRNA sequences used in this study

Movie S1

1. **Movie S1:** Electron tomography of an invasive protrusion in a *flam*$^{\Delta 412\text{-}St2}$ follicle

Data S1 to S2

1. **Data S1:** High resolution spatial localization of capsid accumulations and invasive protrusions at the oocyte-soma interface in *flam*$^{\Delta 412\text{-}St2}$ follicle (same image as fig. S9, left)
2. **Data S2:** High resolution oocyte-soma interface in control follicle (same image as fig. S9, right)

bioRxiv preprint doi: https://doi.org/10.1101/2025.03.14.642691; this version posted March 16, 2025. The copyright holder for this preprint (which was not certified by peer review) is the author/funder, who has granted bioRxiv a license to display the preprint in perpetuity. It is made available under aCC-BY 4.0 International license.

Materials and Methods

Flies

Drosophila melanogaster husbandry and strains

Flies were grown at 25℃ with 12h dark/light cycles under standard laboratory conditions. For dissections of ovaries, 1-3 days old flies post-eclosion were transferred to cages with daily changed apple juice plates and yeast paste for 2 days. Ovaries were collected and stored in ice-cold PBS until fixation for up to 30 minutes after dissection. All fly genotypes are described in Supplementary table 2.

Somatic piRNA pathway knockdown flies

Tissue-specific knockdown of the piRNA/PIWI pathway was obtained by crossing *traffic jam-GAL4*, to UAS-*vreteno*^{GD} flies (somatic piRNA pathway knockdown) or UAS-*arrestin2*^{GD} (control knockdown) (Dietzl et al., 2007; Olivieri et al., 2010). *Tj-GAL4* was recombined with *UAS-myrGFP* (myristoylated GFP) to label somatic membranes in smFISH and in immunofluorescence experiments.

Generation of *flam*$^{\Delta 412\text{-}St2}$ flies

To delete the single full-length insertion of *412* from the *flamenco* locus, gRNAs targeting unique regions upstream (ggatctatttcctggacac) and downstream (ggtggcttcacaaaacacga) of *412-Stalker2* were cloned into a pDCC6b plasmid (Gokcezade et al., 2014) and co-injected with an HDR donor oligo (IDT) to introduce FRT sites into embryos containing the *iso-1* X-chromosome. Successful targeting events were identified by PCR and confirmed by Sanger sequencing.

FRT donor upstream sequence:
ggtccaaaaaccttctagcttgccctctggaccaaactggatctatttcctgGAAGTTCCTATaCtttctagaGAATAGGAACTTCg
GAATAGGAACTTCacaggaccaaagtcgcgcgcgttctcacaactcgtatttagttttcgcaatctacc

FRT donor downstream sequence:

ggtatgttaagtttataatattttacgccaatttccgcaagccggtggcttcacaaaacaGAAGTTCCTATTCcGAAGTTCCTATTCtc
tagaaaGtATAGGAACTTCgacggagtaacttttaagaactctttattgagtagagcaagtgctgttgcttatgagg

We generated female flies heterozygous for both FRT insertions in *trans* and carrying a *nanos-flp* construct on the 2nd chromosome (Kaushal et al., 2021). This resulted in the FRT-Flp mediated deletion of a 15.5kb region between the two FRT sites in the progeny of those females. Individual offspring were screened by PCR for this deletion and verified by Sanger sequencing. Two independent lines (#1 and #2) were retained for further experiments. Control flies (*FRT*) were generated by retaining the integrated FRT site without the Flp recombination, representing genetically identical flies except the *flamenco* deletion. Both *flam*$^{\Delta 412\text{-}St2}$ and the control flies were crossed to *iso-1/CyO* flies to isogenize the 2nd chromosome (*flam*$^{\Delta 412\text{-}St2}$;*iso-1/iso-1*;;) and to GFP-labelled Myosin II (*zip*) to label membranes.

Generation of *flam*$^{\Delta 412\text{-}St2}$; p(*flam*)-PR412sORF2-HA flies

A piRNA-resistant *412 sORF2* (PR412sORF2) sequence was designed by altering wobble positions of the original *412 sORF2* consensus sequence, and synthesized as a G-block (IDT). The PR412sORF2 was tagged with a short linker and 3xHA at the C-terminal and cloned into the *flamenco* reporter vector (Senti

bioRxiv preprint doi: https://doi.org/10.1101/2025.03.14.642691; this version posted March 16, 2025. The copyright holder for this preprint (which was not certified by peer review) is the author/funder, who has granted bioRxiv a license to display the preprint in perpetuity. It is made available under aCC-BY 4.0 International license.

et al., 2023) by replacing the *lacZ* ORF, downstream of a putative ~4 kb *flamenco* promoter to drive constitutive expression in somatic follicle cells. The construct was integrated into *attp40* and balanced flies were subsequently crossed to *flam*$^{\Delta 412\text{-}St2}$ and *FRT* control flies.

LacZ LTR reporter transgene and functional analysis

The 514 bp LTR of *412* was cloned upstream of a *lacZ* gene and integrated into *attP40* as in (Senti et al., 2023). The constructs were combined with piRNA pathway knockdown RNAi lines *UAS-vreteno*^{GD} and *UAS-arrestin2*^{GD} for somatic piRNA pathway knockdown, or with shRNA-lines targeting *aub+ago3* or *white* (control) for germline piRNA pathway knockdown. Ovaries from progeny flies were dissected and stained with blue precipitate-forming X-Gal in chromogenic β-Galactosidase assays as previously described (Handler et al., 2011). The samples were imaged on an Axio Imager.Z2 widefield microscope with an Axiocam 506 color camera for X-gal stainings.

RNA experiments

poly(A)-enriched RNA-seq of ovaries

Sequencing of polyA RNA-seq libraries of ovaries from *Tj> vreteno*^{GD} and *Tj> arrestin2*^{GD} flies was performed in (Senti et al., 2023) and analyzed in this manuscript for specific TEs including the MDG1 group. For *flam*$^{\Delta 412\text{-}St2}$ RNA-seq, total RNA was extracted with TRIzol (Invitrogen) from ovaries of 3 independent replicates of *flam*$^{\Delta 412\text{-}St2}$;*iso-1/iso-1*;; line #1 and line #2 flies, and 4 independent replicates from control *FRT*;*iso-1/iso-1*;; flies (total 10 samples). RNA was treated with DNase I (ThermoFisher Scientific) and purified using RCC25 kit (Zymo Research) with on-column Zymo DNaseI according to kit instructions. Eluted RNA was polyA-selected twice using magnetic Dynabeads Oligo $(dT)_{25}$ (ThermoFisher Scientific) and subjected to NEBNext Ultra II strand-specific RNA-seq libraries preparation (NEB). Quality control of the RNA and the final libraries was evaluated using Agilent Fragment Analyzer and Qubit fluorometer (ThermoFisher Scientific). Libraries were sequenced using NovaSeqX1B. Reads were mapped, quantified and visualized as described in (Baumgartner et al., 2022).

Small RNA-seq libraries of ovaries

Small RNA-seq libraries of two independent replicates of 5 pairs of ovaries from *flam*$^{\Delta 412\text{-}St2}$;;; line #1 and line #2 and control *FRT*;;; flies were generated as previously described (Grentzinger et al., 2020), by isolating Argonaute-sRNA complexes using TraPR ion exchange spin columns. 3′ adaptors containing six random nucleotides plus a 5 nt barcode on their 5′ end and 5′ adaptors containing four random nucleotides at their 3′ end were subsequently ligated to the small RNAs before reverse transcription, PCR amplification, and sequencing on an Illumina NovaSeqX1B. Raw reads were trimmed and mapping was done as in (Baumgartner et al., 2022).

Long-read direct RNA-sequencing of OSCs and ovaries

OSCs were grown as in (Niki et al., 2006; Saito et al., 2009) and transfected with siRNA targeting PIWI and control siRNA targeting GFP using Cell Line Nucleofector kit V (Amaxa Biosystems) with the program T-029. siRNA sequences are found in Supplementary table 2. After two days the cells were transfected again and harvested two days later. For ovaries of *Tj> vreteno*^{GD} and *Tj>arrestin2*^{GD} flies, approximately

bioRxiv preprint doi: https://doi.org/10.1101/2025.03.14.642691; this version posted March 16, 2025. The copyright holder for this preprint (which was not certified by peer review) is the author/funder, who has granted bioRxiv a license to display the preprint in perpetuity. It is made available under aCC-BY 4.0 International license.

20 flies per sample were dissected, the ovaries washed with PBS, flash-frozen, and stored at -80°C until processing.

RNA was extracted by homogenizing the samples in TRIzol, and performing phase separation with chloroform, followed by centrifugation at 4816 g for 10 minutes. RNA was precipitated with isopropanol, centrifuged at 20,000 g for 1 hour at 4°C, washed with 75% ethanol, air-dried, and dissolved in nuclease-free water. Poly(A) RNA was enriched using oligo(dT) Dynabeads, with RNA incubated at 80°C, snap-cooled, and bound to beads in binding buffer. After washing, poly(A) RNA was eluted in water at 80°C and quantified using Qubit RNA BR Assay. Libraries were prepared using the ONT RNA002 Direct RNA Library Prep Kit (SQK-002) following the manufacturer's instructions. Briefly, the ONT RNA adapter was ligated using the NEBNext Quick Ligation Kit, followed by reverse transcription with SuperScript III. After purification with Ampure beads, a second ligation step added the ONT sequencing adapter. Following a final cleanup, libraries were quantified using the Qubit DNA HS Assay, and ~400 ng of library was used for sequencing. Sequencing was performed on ONT R9 flow cells using MinION Mk1b sequencer. Basecalling was conducted with Dorado v0.8+98ff765, with polyA-tail detection enabled. For TE 3′-end detection, reads with a detected polyA tail were aligned in splice mode using minimap2 to the TE consensus sequences, truncated to their 3′ ends, and analyzed with bedtools genomecov to generate histograms. Visualization of long reads data was done with Tablet (Milne et al., 2013).

smFISH of ovaries

smFISH probes targeting the *gag* region in *412, gag-pol* in *Mdg1* and *sORF2-gag-pol* in *Stalker2* sequences were ordered as Stellaris probes for *412* and *Mdg1* (Biosearch Technologies) or as DNA oligos to be labeled in-house (*Stalker2*, IDT) (Gaspar et al., 2017). Probe sequences are listed in Supplementary table 2. smFISH was performed as in (Baumgartner et al., 2022). Briefly, ovaries were dissected, washed and fixed in 4% paraformaldehyde in PBX (PBS with 0.3% Triton-X) at room temperature for 20 minutes. The ovaries were washed and permeabilized with PBX, equilibrated with FISH wash buffer (10% formamide in 2x SSC) and incubated overnight in 50 µL FISH hybridization buffer (100 mg/mL dextran sulfate and 10% formamide in 2x SSC) with 0.5 µL FISH probe at 37℃. The ovaries were then washed 3x in wash buffer and counterstained with DAPI in 2xSSC, washed with 2XSSC and mounted for imaging.

Imaging

All samples for imaging were mounted on slides using ProLong Diamond mounting medium (Invitrogen) and imaged as confocal Z-stacks using Olympus IX3 Series (IX83) inverted microscope, equipped with a Yokogawa W1 spinning disk (SD) of 50 µm pinhole size. Olympus lenses UPLSAPO2 10x/0.4, UPLFLN 40x /0.75, and UPLSAPO 100x /1.4 were used for the acquisition. Diode lasers (OBIS LX, Coherent Corp.) of 405, 488, 561, and 640 nm were used at 100% power (less than 1440, 7970, 7200, and 5700 mW respectively) with the following filters for the emission: 447/60, 525/50, 617/73, 685/40nm. Camera exposure times were set to 300 ms for all channels, and images were acquired with a Hamamatsu ORCA-Fusion (C14440-20UP CMOS) camera. Z-sampling was determined by the optimal Nyquist option in OLYMPUS cellSens Dimension 2.3 software, corresponding to 250 nm in all 100x Z-stacks (65 nm/pixel) and 500 nm in all 40x z-stacks (162.5 nm/pixel). Representative images were then deconvolved using Huygens Professional 24 software (SVI). Deconvolution parameters were individually selected, following the recommendations within the software, with SNR values ranging between 10-30, background subtraction

bioRxiv preprint doi: https://doi.org/10.1101/2025.03.14.642691; this version posted March 16, 2025. The copyright holder for this preprint (which was not certified by peer review) is the author/funder, who has granted bioRxiv a license to display the preprint in perpetuity. It is made available under aCC-BY 4.0 International license.

100 and 30 iterations used. Images were visualized with IMARIS 10 software (Oxford Instruments), setting identical color adjustments to experimental sample images and their respective controls, and individual Z-slices were exported as high-quality TIFF images for the manuscript preparation.

Protein experiments

Generation of polyclonal antibodies

The rat polyclonal antibody targeting 412 Gag (raised against the antigen MLKNDDKTNNAADSE) in co-localization experiments was a kind gift from E. Brasset. Newly generated polyclonal antibodies targeting 412 Gag, 412 sORF1 and 412 sORF2 were raised in rabbits as follows: For Gag, the same antigen was synthesized in-house with N-terminal cysteine residue and coupled to KLH. For sORF1, recombinant His-tagged sORF1 protein was expressed in *E. coli* BL21(DE3), purified by Ni-NTA affinity chromatography followed by ion-exchange chromatography. For sORF2, the antigen from the extracellular region (α-sORF2-N, DTKTVDSTANVINKVEVQNAD) and the antigen from the cytoplasmic region (α-sORF2-C, LKKRYRSTANGIDNI) were synthesized in-house with terminal cysteine residue and coupled to KLH. The synthetic peptides and the sORF1 protein were injected into rabbits to generate polyclonal antisera using the Speedy 28 days protocol (Eurogentec) and purified using glycine elutions of the peptide synthesis columns (Gag, sORF2) or of bound recombinant protein (sORF1).

Whole-mount immunofluorescence of ovaries

Immunofluorescence of dissected ovaries was done as in (Baumgartner et al., 2022), and the primary antibodies were used in overnight incubations at 4℃ in the following concentrations: rat anti-Gag (1:1,000), rabbit anti-Gag (1:500), α-sORF1 (1:500), α-sORF2-N (1:250), α-sORF2-C (1:500), rat monoclonal anti-HA (ROAHAHA, Roche) 1:1,000. For labeling F-actin, Alexa Fluor® 488 phalloidin (Invitrogen) was used together with the secondary antibodies at 1:500 and incubated overnight at 4℃. For labeling DNA, DAPI was used at 1:10,000 for 10 minutes at room temperature.

Mass-spectrometry

Sample preparation for mass-spectrometry experiments

Dissected ovaries from somatic piRNA pathway knockdown (*Tj>vreteno*^{GD}*)* and control knockdown *(Tj>arrestin2*^{GD}*)* flies were dissolved in 500 µL methanol and then protein was precipitated by adding 1mL chloroform. After centrifugation (10 min 10,000 rcf) the supernatant was removed. The pellet was lysed in 10M urea 50mM HCl and then 1M TEAB was added to a final concentration of 100mM. For the reduction of the cysteines DTT and for the alkylation of the free cysteines IAA was used. The samples were diluted with 100mM TEAB Buffer to a urea concentration of 6M and digested with LysC with an enzyme-to-protein ration of 1:50 for 3h at 37 ºC. After the pre-digestion with LysC (Wako) the sample was diluted with 100mM TEAB buffer to final urea concentration of 2M. The tryptic digest (Trypsin Gold, Promega) was done with an enzyme-to-protein ration of 1:50 for overnight at 37ºC. After the pre-digestion with LysC (Wako) the sample was diluted with 100mM TEAB Buffer to final urea concentration of 2M. The tryptic digest (Trypsin Gold, Promega) was done with an enzyme-to-protein ration of 1:50 for overnight at 37ºC.

bioRxiv preprint doi: https://doi.org/10.1101/2025.03.14.642691; this version posted March 16, 2025. The copyright holder for this preprint (which was not certified by peer review) is the author/funder, who has granted bioRxiv a license to display the preprint in perpetuity. It is made available under aCC-BY 4.0 International license.

For OSCs, somatic PIWI knockdown was done with siRNAs as for long-read direct sequencing, using siRNA against GFP as a control. For the digest of the sampled the iST 96x Kit (PreOmics GmbH) was used and performed according to the manufacturer's protocol.

NanoLC-MS Analysis

The nano-HPLC system used was an UltiMate 3000 RSLC nano system (Thermo Fisher Scientific, Amsterdam, Netherlands) coupled to a Q Exactive HF-X or an Orbitrap Eclipse Tribrid mass spectrometer (both Thermo Fisher Scientific, Bremen, Germany), equipped with a Proxeon nanospray source (Thermo Fisher Scientific, Odense, Denmark). Peptides were loaded onto a trap column (Thermo Fisher Scientific, Amsterdam, Netherlands, PepMap C18, 5 mm × 300 μm ID, 5 μm particles, 100 Å pore size) at a flow rate of 25 μL min-1 using 0.1% TFA as mobile phase. After 10 min, the trap column was switched in line with the analytical column (Thermo Fisher Scientific, Amsterdam, Netherlands, PepMap C18, 500 mm × 75 μm ID, 2 μm, 100 Å). Peptides were eluted using a flow rate of 230 nl min-1, and a binary 2h or 3h gradient respectively, which results in a total run time of 140 min or 225 min respectively.

The gradient starts with the mobile phases: 98% A (water/formic acid, 99.9/0.1, v/v) and 2% B (water/acetonitrile/formic acid, 19.92/80/0.08, v/v/v), increases to 35% B over the next 120 or 180 min, followed by a gradient in 5 min to 90%B, stays there for 5 min and decreases in 2 min back to the gradient 98%A and 2%B for equilibration at 30 °C.

The Q Exactive HF-X mass spectrometer was operated in data-dependent mode, using a full scan (m/z range 380-1500, nominal resolution of 60,000, target value 1E6) followed by 10 MS/MS scans of the 10 most abundant ions. MS/MS spectra were acquired using normalized collision energy of 28, isolation width of 1.0 m/z, resolution of 30.000 and the target value was set to 1E5. Precursor ions selected for fragmentation (exclude charge states unassigned, 1, 7, 8, >8) were put on a dynamic exclusion list for 60 s. Additionally, the minimum AGC target was set to 5E3 and intensity threshold was calculated to be 4.8E4. The peptide match feature was set to preferred and the exclude isotopes feature was enabled.

The Eclipse was operated in data-dependent mode, performing a full scan (m/z range 375-1500, resolution 120,000, target value 1E6, normalized AGC target 250%) at 3 different compensation voltages (CV-45, -55, -75), followed each by MS/MS scans of the most abundant ions for a cycle time of 1 sec per CV. MS/MS spectra were acquired using an isolation width of 1 m/z, AGC target value of 30,000, normalized AGC target 300%, minimum intensity of 50,000, maximum injection time 15 ms, activation type.

HCD with a collision energy of 30 %, using the Iontrap for detection in the scan mode Turbo. Precursor ions selected for fragmentation (included charge states 2-6) were excluded for 40 s. The monoisotopic precursor selection filter and exclude isotopes feature were enabled.

Mass-spectrometry data processing

For peptide identification, the RAW-files were loaded into Proteome Discoverer (version 2.5.0.400, Thermo Scientific). All hereby created MS/MS spectra were searched using MSAmanda v2.0.0.16129, Engine version v2.0.0.16129 (Dorfer et al., 2014). The RAW-files were searched against the *Drosophila melanogaster* database of Flybase, version r6.36 (22,226 sequences; 20,310,919 residues) and a TE database, which includes all BDGP consensus sequences (Kaminker et al., 2002) translated in six reading frames with a minimum open reading frame length of 40 amino acids, supplemented with common

bioRxiv preprint doi: https://doi.org/10.1101/2025.03.14.642691; this version posted March 16, 2025. The copyright holder for this preprint (which was not certified by peer review) is the author/funder, who has granted bioRxiv a license to display the preprint in perpetuity. It is made available under aCC-BY 4.0 International license.

contaminants, using the following search parameters: Iodoacetamide derivative on cysteine was set as a fixed modification, oxidation on methionine and deamidation on asparagine and glutamine were set as variable modifications. The peptide mass tolerance was set to ±5 ppm and the fragment mass tolerance to ±8 ppm for the QEx-HFX-measurement, as well as ±5 ppm and fragment mass tolerance of ±500 mmu for the Eclipse-measurement. The maximal number of missed cleavages was set to 2, using tryptic enzymatic specificity. The result was filtered to 1 % FDR on protein level using Percolator algorithm (Käll et al., 2007) integrated in Thermo Proteome Discoverer.

The localization of the post-translational modification sites within the peptides was performed with the tool ptmRS, based on the tool phosphoRS (Taus et al., 2011).

Protein areas have been computed in IMP-apQuant (Doblmann et al., 2019) by summing up unique and razor peptides. Resulting protein areas were normalized using iBAQ (Schwanhäusser et al., 2011) and sum normalization was applied for normalization between samples.

Electron microscopy and tomography

Preparation for ultrastructural preservation and tomography

Drosophila flam$^{\Delta 412\text{-}St2}$;*iso-1* and *FRT;iso-1* ovaries were dissected in ice-cold PBS and mounted in a 200µm recess of an aluminum carrier with a diameter of 3mm, covered with the flat side of a carrier, and immediately high pressure frozen in a HPF Compact 01 (Engineering Office M. Wohlwend GmbH, Switzerland). To avoid air bubbles, 5% BSA in Sörensen phosphate buffer was used as filling material. Subsequently the samples were processed in a Leica AFS-2 automated freeze substitution unit (Leica Microsystems, Austria), following this protocol: 60h at –90°C, warm up at a rate of 2°C per hour to –54°C, 18h at –54°C, warm up at a rate of 5°C per hour to –24, 15h at –24°C, warm up at a rate of 6°C per hour to 20°C, 5h at 20°C. Over this 5d-period, the frozen water in the samples was substituted with acetone containing 1% osmium tetroxide, 0.2% uranyl acetate and 5% water (UA from Merck, Germany; OsO4 from EMS, USA). The dehydrated samples were washed 3 times in anhydrous acetone at RT. Samples were infiltrated with a medium hard mixture of Epoxy resin (Agar 100 from Agar Scientific, UK), in a graded series of acetone and resin (2:1, 1:1, 1:2 for 1h each), followed by pure resin for a few hours and polymerization in the oven for 2d at 60°C.

TEM ultrastructure imaging of epon-fixed samples

The obtained resin blocks were cut using a Leica UCT ultramicrotome (Leica Microsystems, Austria) at a nominal thickness of 50-70nm and the sections were picked up on 100mesh copper grids or slot-grids (Agar Scientific, UK), previously coated with a self-made Formvar® support film (Formvar powder from Agar Scientific, UK). Sections obtained from high pressure freezing/freeze substitution, did not need any further contrasting. Grids were then examined in a FEI Morgagni 268D transmission electron microscope, operated at a high tension of 80kV (FEI/Thermo Fisher, The Netherlands). Digital images were acquired using a Megaview III CCD camera (Olympus-SIS, Germany). For quantification of capsid diameter, 120 capsids from different images taken at different time points were measured manually and the average diameter represents the most frequently observed capsid size.

bioRxiv preprint doi: https://doi.org/10.1101/2025.03.14.642691; this version posted March 16, 2025. The copyright holder for this preprint (which was not certified by peer review) is the author/funder, who has granted bioRxiv a license to display the preprint in perpetuity. It is made available under aCC-BY 4.0 International license.

Electron Tomography

Epon-fixed samples were sectioned into 250 nm sections, and a concentrated solution of 10 nm gold beads (Aurion, The Netherlands) was applied in a single drop and incubated for a period of three minutes. Double-axis tilt series were imaged using a Tecnai G2 20 microscope equipped with an Eagle 4 k HS CCD camera (FEI, the Netherlands). The microscope was operated at 200 kV and the SerialEM software (Mastronarde, 2005) was employed for data acquisition. To prevent shrinkage during the acquisition of tilt series data, the section was subjected to low-magnification cooking for a period of 20 minutes prior to data acquisition. Dual axis tilt series were recorded at 1° increments with a tilt range of -57°/+52° and -60°/+59°, respectively. The raw data were processed into tomograms using the IMOD software (Mastronarde & Held, 2017).

Cryo-immunoEM - Tokuyasu method

Drosophila flam$^{\Delta 412\text{-}St2}$;*iso-1* and *FRT;iso-1* ovaries were dissected in ice-cold PBS and fixed in 2% paraformaldehyde and 0.2% glutaraldehyde (both EM-grade, EMS, USA) in 0.1 M PHEM buffer (pH 7) for 2h at RT, then overnight at 4°C. The fixed ovaries were embedded in 12% gelatin and cut into 1 mm^3 blocks which were immersed in 2.3 M sucrose overnight at 4°C. These blocks were mounted onto Leica specimen carrier (Leica Microsystems, Austria) and frozen in liquid nitrogen. With a Leica UCT/FCS cryo-ultramicrotome (Leica Microsystems, Austria) the frozen blocks were cut into ultra-thin sections at a nominal thickness of 70nm at -110°C. A mixture of 2% methylcellulose (25 centipoises) and 2.3 M sucrose in a ratio of 1:1 was used as a pick-up solution. Sections were picked up onto 200 mesh Ni grids (Gilder Grids, UK) with a carbon coated formvar film (Agar Scientific, UK). Fixation, embedding and cryo-sectioning as described by (Tokuyasu, 1973). Prior to immunolabeling, grids were placed on plates with solidified 2% gelatine and warmed up to 37 °C for 20 min to remove the pick-up solution. After quenching of free aldehyde-groups with glycine (0.1% for 15 min), a blocking step with 1% BSA (fraction V) in 0.1 M Sörensen phosphate buffer (pH 7.4) was performed for 40 min. The grids were incubated in primary antibody (α-Gag and α -sORF2-C), diluted 1:50 in 0.1 M Sörensen phosphate buffer over night at 4°C, followed by a 2h incubation in the secondary antibody, a goat-anti-rabbit antibody coupled with 6 nm gold particles (GAR 6 nm, Aurion, The Netherlands), diluted 1:20 in 0.1 M Sörensen phosphate buffer, performed at RT. The sections were stained with 4% uranyl acetate (Merck, Germany) and 2% methylcellulose in a ratio of 1:9 (on ice). All labeling steps were done in a wet chamber. The sections were inspected using a FEI Morgagni 268D TEM (FEI/Thermo Fisher, The Netherlands) operated at 80kV. Electron micrographs were acquired using a Megaview III CCD camera (Olympus-SIS, Germany).

Computational analysis

Annotation and phylogenetic analysis of LTR retrotransposon sequences

Consensus sequences and annotated features (LTRs, *gag* and *pol* ORFs, sORFs) for all *D. melanogaster* LTR retrotransposons studied in this work were collected from the BDGP v.9.4.1 database (Kaminker et al., 2002), Repbase (Bao et al., 2015) and primary literature (Avedisov et al., 1990; Cherkassova et al., 1991; Costas et al., 2001; Makarova, 1997; Mugnier et al., 2005; Yuki et al., 1986). *sORF1* and *sORF2* were identified as predicted 60-130 amino acids long ORFs, positioned between the 5' LTR and *gag* and in the same orientation as *gag*. The absence of intact *env* ORF was determined by measuring the distance

bioRxiv preprint doi: https://doi.org/10.1101/2025.03.14.642691; this version posted March 16, 2025. The copyright holder for this preprint (which was not certified by peer review) is the author/funder, who has granted bioRxiv a license to display the preprint in perpetuity. It is made available under aCC-BY 4.0 International license.

between the end of *pol* and the start of the 3' LTR for the MDG1 group retrotransposons. The phylogenetic tree shown in Fig. 1A was generated by aligning the *pol* sequences from the protease domain to and including the conserved integrase domain as in (Senti et al., 2023), and visualized with iTol v6 (Letunic & Bork, 2021). For *Blood* the consensus sequence in Repbase and BDGP shows only the possible translation of the first 11 amino acids of sORF2, however the sORF2 of *Blood* was determined based on analysis of five heterochromatic *Blood* insertions which show an intact sORF2, but have a defective *gag*, *pol* or interrupted sequence by "nested" TEs.

Flamenco enrichment of TE groups analysis

Non-overlapping genomic windows of 100 kb were generated from the *Drosophila melanogaster* reference genome (dm6), excluding mitochondrial and Y chromosome sequences. To precisely capture the *flamenco* locus, a TE-rich region on the X chromosome (*chrX: 21,631,891–21,931,891*), windows were centered to align exactly with this region. TE content within each window was quantified using RepeatMasker annotations (Smit, A.F.A. Hubley, R., & Green, P., 2013), irrespective of strand orientation. Windows with >50% TE content were classified as heterochromatic tiles and retained for further analysis. TE content was further stratified by strand and TE category (e.g., LTR, LINE, DNA) and visualized in Fig. 1C. Statistical significance for the enrichment of specific TE groups, such as the MDG1 group, was assessed by calculating a z-score and an empirical p-value (*e.g.*, MDG1: $z = 24$, p = 1.45 × 10-2).

Alphafold3 predictions

AlphaFold3 (Abramson et al., 2024) was used to predict protein structures of sORF1 and sORF2 and structures were analyzed using UCSF ChimeraX (Pettersen et al., 2021).

Multiple sequence alignment and sORF2/FAST-like structural features prediction

Amino acid sequences of proteins were aligned using MAFFT (Madeira et al., 2024) and visualized with Jalview (Waterhouse et al., 2009). Manual adjustments were introduced to clarify demonstrated or presumed structural and functional motifs characteristic of FAST proteins. Color scheme is according to ClustalX conservation criteria. The transmembrane domain and extracellular/cytoplasmic topology were predicted using TMHMM (Krogh et al., 2001). N-myristoylation prediction was done with GPS-Lipid for the initial identification of sORF2 proteins in *Drosophila* (Xie et al., 2016), and for all other analyses determined by presence of the consensus MGxxxS/T motif. A polybasic region was identified by having 3 or more K or R amino acids within the first 15 amino acids immediately following the transmembrane domain. Amphipathic helices were predicted and hydrophobic moment <μH> calculated using Heliquest (Gautier et al., 2008).

Identification of insect sORF2/FAST-like candidates

A database of 8,417 consensus sequences of *Gypsy* LTR retroviruses in arthropods was downloaded from Repbase (March 2024). All interior sequences were translated in all six reading frames and screened for sORF2-like proteins using the following criteria: ORF size between 60-210 amino acids (180-630 nt); a single transmembrane domain predicted by TMHMM 2.0c and Phobius 1.01 (Käll et al., 2004); N-terminal extracellular (NONCYTO) and C-terminal intracellular (CYTOPLASMIC) topology ; an ectodomain containing an N-myristoylation motif (MGxxxS/T) with a length between 10-50 amino acids ; and a cytoplasmic region smaller than 150 amino acids. This screening identified 105 candidate sequences, which

bioRxiv preprint doi: https://doi.org/10.1101/2025.03.14.642691; this version posted March 16, 2025. The copyright holder for this preprint (which was not certified by peer review) is the author/funder, who has granted bioRxiv a license to display the preprint in perpetuity. It is made available under aCC-BY 4.0 International license.

were further analyzed for their genomic coordinates within the Repbase consensus sequence (fig. S10) and the taxonomic classification of the arthropod genomes, visualized with iTol (Figure 5B). Alignments and feature annotations (e.g. polybasic, amphipathic motifs) were performed as described above.

Genome-wide scanning of TE insertions in select genomes

To identify individual TE insertions, RepeatMasker version 4.1.7-p1 (Smit, A.F.A. Hubley, R., & Green, P., 2013) was used to screen LTR retrotransposons in the following genomes with custom TE consensus sequence libraries: *D. melanogaster* dm6 (GCF_000001215.4, Dfam database (Storer et al., 2021) using RepeatMasker default parameters -species 7227 rmblastn version 2.14.1 + FamDB: CONS-Dfam_3.8), *A. aegyptii* L5 (GCF_002204515.2) with TE library from (Daron et al., 2024). For *S. oryzae* 2.0 (GCF_002938485.1) TE annotations were taken from (Parisot et al., 2021). Genomic nucleotide sequences were screened for sORF2/FAST-like candidates as described for the Repbase consensus sequences. Resulting candidates were filtered for minimal overlap 20 nt with any RepeatMasker predicted repetitive regions. To match the individual insertions to the identified sORF2/FAST-like candidates from Repbase consensus sequences, BLAST searches on the protein sequences were performed with default parameters, and results were filtered by an E-value cutoff of 1e-10.

Identification of sORF2/FAST like proteins in non-enveloped viruses

The 105 sORF2/FAST-like amino acid sequences identified in insect LTR retrotransposons were aligned using PRANK (Löytynoja, 2014) and an HMM profile was constructed using HMMER v3.4 (Eddy, 2011). This HMM profile was used to search the NCBI nr database (January 2025) for viral sequences, with human and non-human viruses analyzed separately. For non-human viruses, 56 hits passed the E-value threshold of 0.05, including 39 sequences from picornaviruses and 2 from nackednaviruses. All picornavirus genomes identified with this search that were between 1,500 and 20,000 nt in length are shown in Supplementary Figure S11. Select sequences were aligned and shown in Figure 5E, including Goose megrivirus A (NC_033793.1), Duck megrivirus A2 (KC663628.1), Swan megrivirus A, Chicken megrivirus C (KF961187.1), Turkey melegrivirus A (KF961188.1), Peafowl picornavirus sp. (MT138340.1), and Chickadee poecivirus A1 (KU977108.1).

For nackednaviruses, all 13 genome sequences lacking *preS/S* gene were extracted from Supplementary file S1 of (Lauber et al., 2017) and shown as genome annotations in Supplementary Figure S11. sORF2/FAST-like features were predicted using the same criteria as for insect LTR retrotransposon candidates. Select sequences were aligned and shown in Fig. 5F (African cichlid ACNDV, European eel EENDV, Rockfish RNDV, Astatotilapia ANDV, Rainwater killifish KNDV-Lp-1).

bioRxiv preprint doi: https://doi.org/10.1101/2025.03.14.642691; this version posted March 16, 2025. The copyright holder for this preprint (which was not certified by peer review) is the author/funder, who has granted bioRxiv a license to display the preprint in perpetuity. It is made available under aCC-BY 4.0 International license.

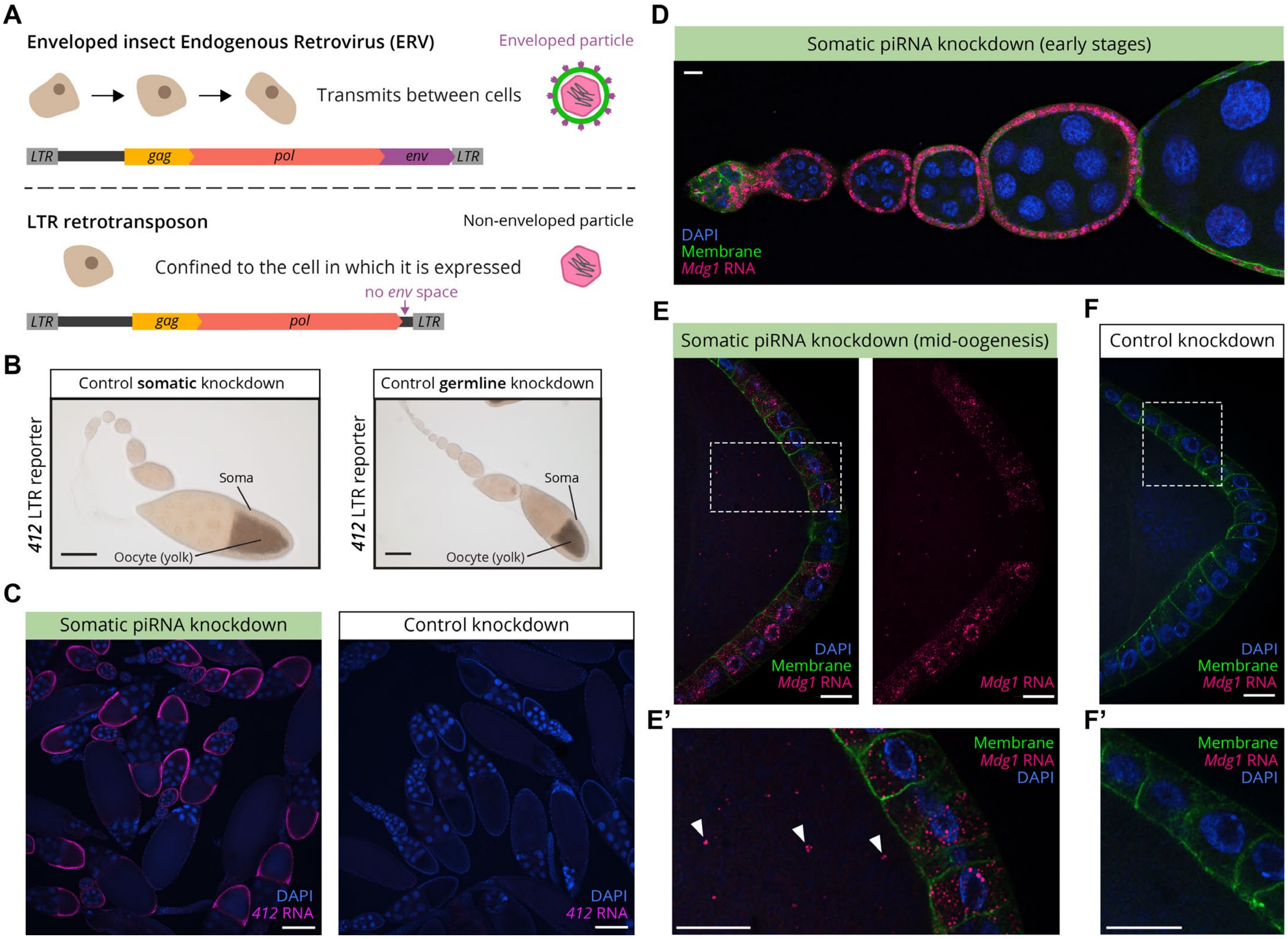

Supplementary Figure 1: Robust somatic expression and oocyte transmission of MDG1 retrotransposons. **(A)** Schematic comparison of insect ERV and LTR retrotransposon genome organization. Insect ERVs encode an Envelope protein (purple) that enables cell-to-cell transmission, while LTR retrotransposons lack an *env* gene and are typically confined to the cell in which they are expressed. **(B)** Related to Figure 1E - control knockdowns of the transcriptional *412 LTR-lacZ* reporter, left: somatic piRNA pathway knockdown (*Tj>Gal4,arr2-RNAi*), right: germline piRNA pathway knockdown (*MTD>Gal4,wsh-RNAi*). Scale bar: 100 µm. **(C)** Left: Overview of somatic piRNA pathway knockdown (*Tj>Gal4,vret-RNAi*) sample showing *412* smFISH (magenta) signal in somatic follicle cells across multiple ovarioles. Right: overview of *412* smFISH signal in control knockdown (*Tj>Gal4,arr2-RNAi*) ovarioles. **(D-F)** smFISH staining of **(D)** early and **(E)** mid-oogenesis follicles for LTR retrotransposon *Mdg1* (pink), recapitulating the soma-to-oocyte transfer seen for additional MDG1 group retrotransposons in the somatic piRNA pathway knockdown background (*Tj>Gal4,vret-RNAi*, left) whereas in **(F)** control ovaries *Mdg1* is not expressed (*Tj>Gal4,arr2-RNAi*). White arrowheads denote RNA signals within the oocyte. In (D-F) somatic membranes are marked by myristoylated GFP. Scale bars (C-F): 10 µm.

bioRxiv preprint doi: https://doi.org/10.1101/2025.03.14.642691; this version posted March 16, 2025. The copyright holder for this preprint (which was not certified by peer review) is the author/funder, who has granted bioRxiv a license to display the preprint in perpetuity. It is made available under aCC-BY 4.0 International license.

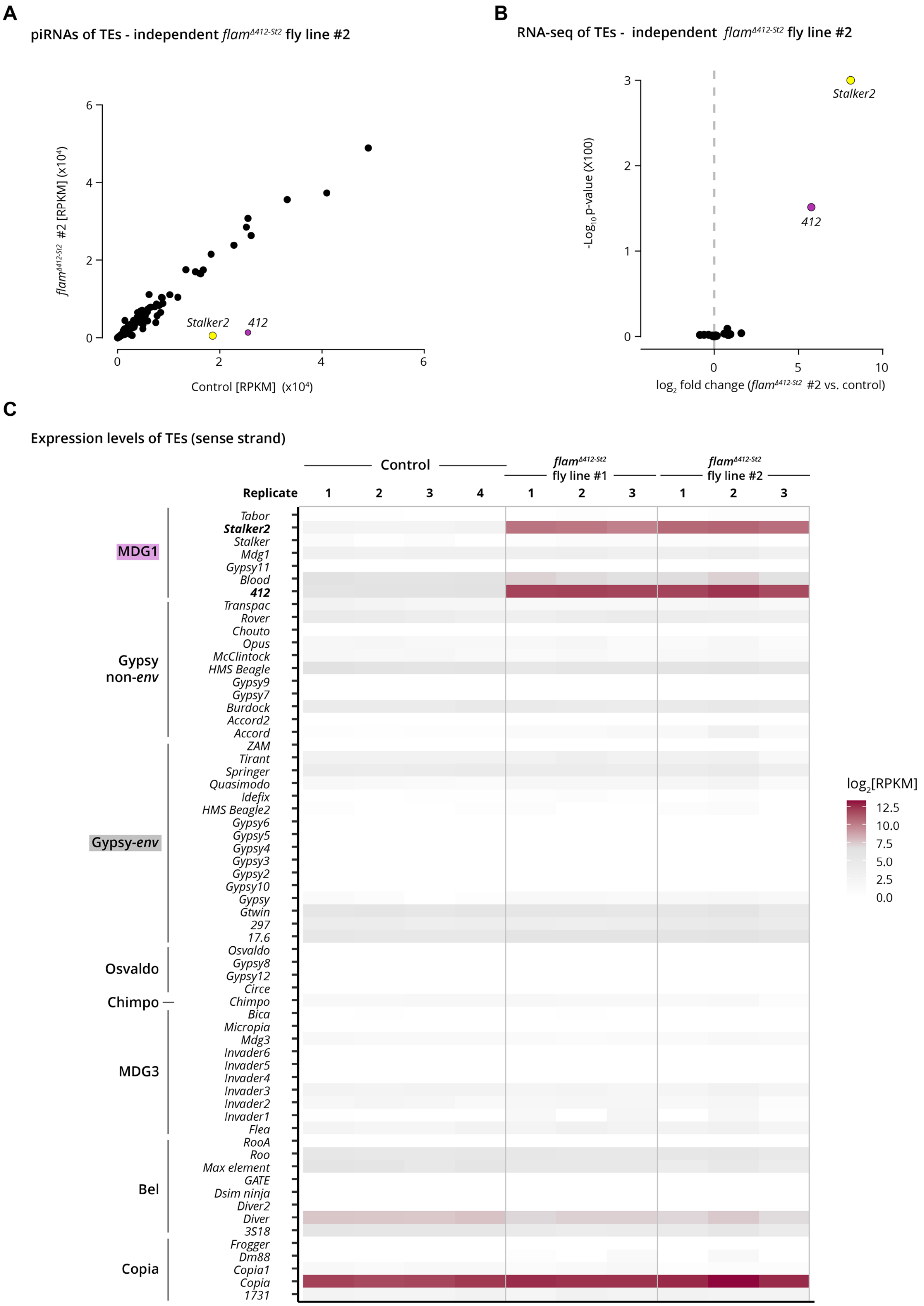

bioRxiv preprint doi: https://doi.org/10.1101/2025.03.14.642691; this version posted March 16, 2025. The copyright holder for this preprint (which was not certified by peer review) is the author/funder, who has granted bioRxiv a license to display the preprint in perpetuity. It is made available under aCC-BY 4.0 International license.

Supplementary Figure 2: RNA expression of TEs in *flam*$^{\Delta 412\text{-}St2}$ ovaries. **(A)** Small RNA sequencing of piRNAs from *flam*$^{\Delta 412\text{-}St}$ ovaries (homozygous mutant line #2), compared to control ovaries (genetically identical except for the targeted *flamenco* deletion). Each datapoint represents the number of antisense reads mapped to a TE consensus sequence, normalized as reads per kilobase of TE per million mapped microRNAs (RPKM). **(B)** Volcano plot of whole transcriptome poly(A)-tailed RNA-seq, from *flam*$^{\Delta 412\text{-}St2}$ ovaries(homozygous mutant line #2), compared to control ovaries (n=3). Each datapoint represents the number of reads mapped to a TE consensus sequence. **(C)** Heatmap of expression levels [RPKM] for 62 LTR retrotransposons across three replicates of *flam*$^{\Delta 412\text{-}St}$ ovaries (two independent mutant lines) and four replicates of control ovaries. Values between 0 and 1 are rounded to 1 and represented as white squares. *Copia*, which does not encode for an Envelope, is known to be constitutively highly expressed in ovaries (Klumpe et al., 2025).

bioRxiv preprint doi: https://doi.org/10.1101/2025.03.14.642691; this version posted March 16, 2025. The copyright holder for this preprint (which was not certified by peer review) is the author/funder, who has granted bioRxiv a license to display the preprint in perpetuity. It is made available under aCC-BY 4.0 International license.

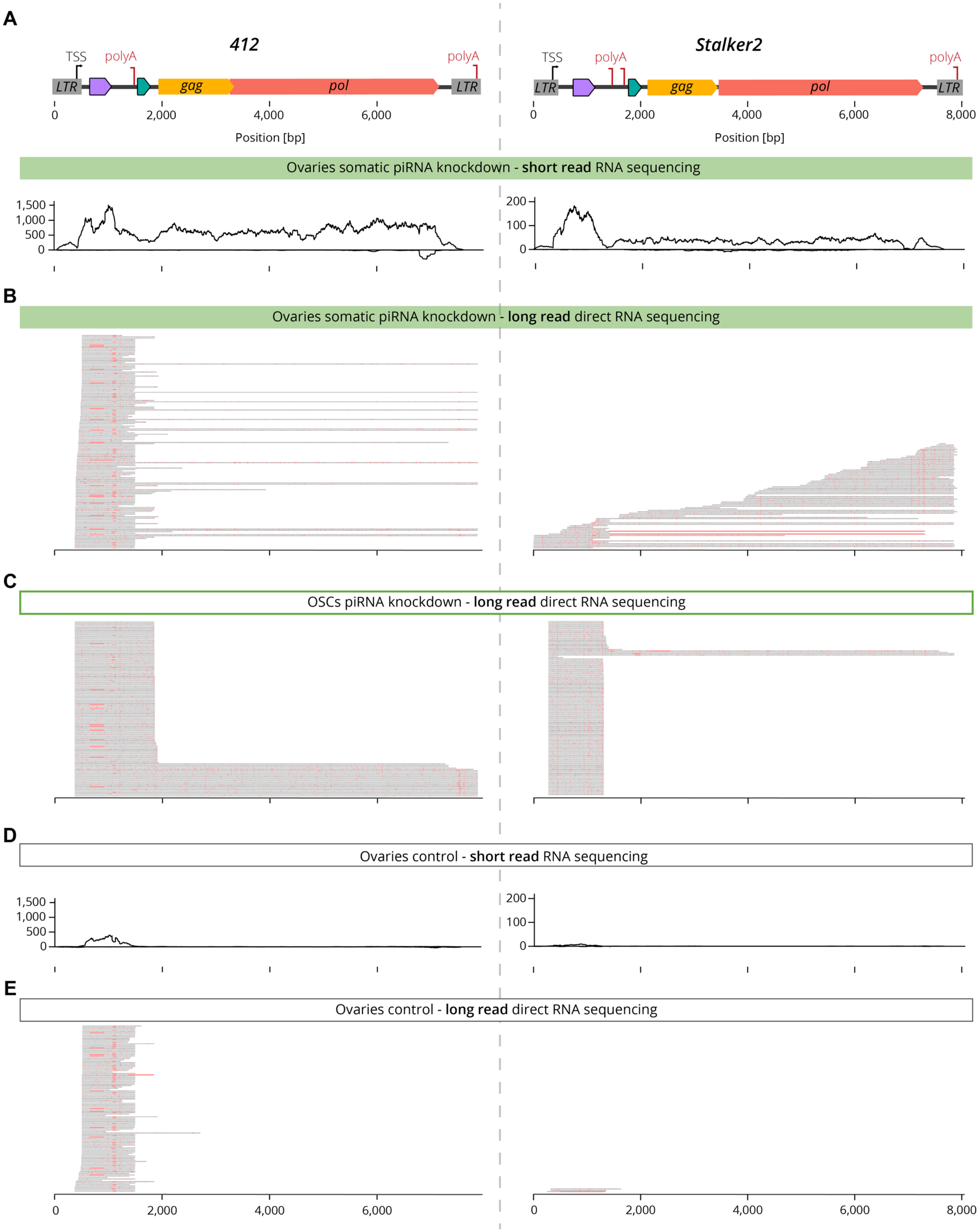

bioRxiv preprint doi: https://doi.org/10.1101/2025.03.14.642691; this version posted March 16, 2025. The copyright holder for this preprint (which was not certified by peer review) is the author/funder, who has granted bioRxiv a license to display the preprint in perpetuity. It is made available under aCC-BY 4.0 International license.

Supplementary Figure 3: *412* and *Stalker2* produce long and short transcripts. For (A-E), *412* data is shown on the left and *Stalker2* is shown on the right. For (B,C,E) partial subsets of long-read direct RNA sequencing reads are shown, with reads selected to show both long and short isoforms, and sorted according to the first mapped nucleotide of the read. Variant regions deviating from consensus are shown in red. **(A)** Coverage plots of short-reads RNA-sequencing of one representative replicate from somatic piRNA pathway knockdown ovaries (*Tj>Gal4,vret-RNAi*) sample. Y-axis is number of reads mapped to consensus sequences, normalized as reads per kilobase of TE per million mapped reads (RPKM). **(B)** Subset of long-read direct RNA sequencing reads, showing individual reads mapped to the consensus sequence, of the same genotype as in (A). **(C)** Subset of long-read direct RNA sequencing reads from somatic piRNA pathway knockdown in OSCs (treated with siRNA targeting Piwi). **(D)** Coverage plots of short-reads RNA-sequencing of one representative replicate from control knockdown ovaries (*Tj>Gal4,arr2-RNAi*) sample. Y-axisas in (A). **(E)** Subset of long-read direct RNA sequencing reads, showing individual reads mapped to the consensus sequence, of the same genotype as in (D).

bioRxiv preprint doi: https://doi.org/10.1101/2025.03.14.642691; this version posted March 16, 2025. The copyright holder for this preprint (which was not certified by peer review) is the author/funder, who has granted bioRxiv a license to display the preprint in perpetuity. It is made available under aCC-BY 4.0 International license.

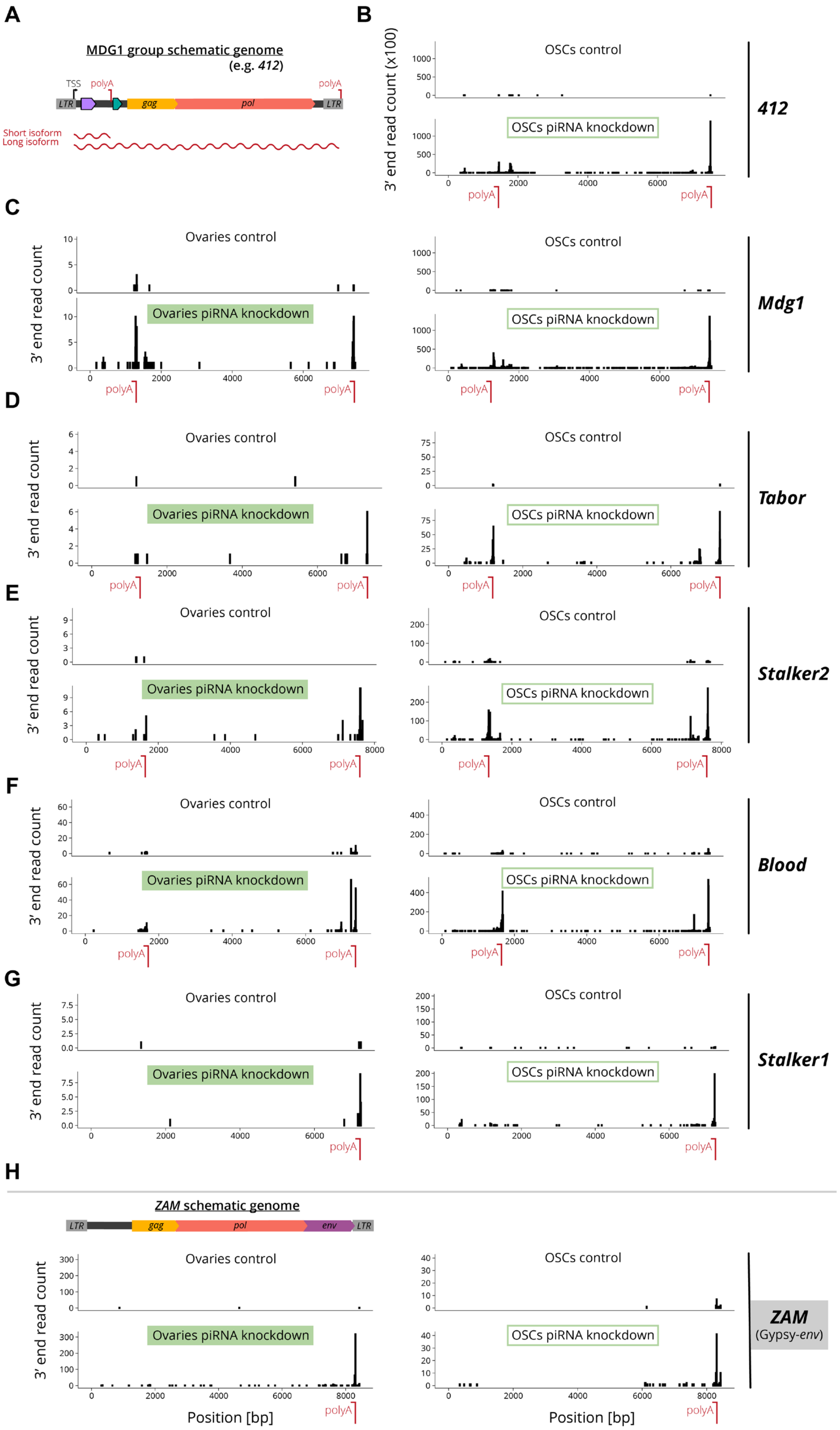

bioRxiv preprint doi: https://doi.org/10.1101/2025.03.14.642691; this version posted March 16, 2025. The copyright holder for this preprint (which was not certified by peer review) is the author/funder, who has granted bioRxiv a license to display the preprint in perpetuity. It is made available under aCC-BY 4.0 International license.

Supplementary Figure 4: Premature transcription termination sites are found in most MDG1 retrotransposons. (A) Schematic representation pf MDG1 LTR retrotransposon genomes, showing ORFs (colored arrows), LTRs (grey boxes), transcription termination sites ("polyA"), and wavy lines representing the short and long isoforms produced based on the polyA site that is predominantly used. **(B-H)** Mapping of 3' ends of long-read direct RNA-sequencing reads corresponding to the consensus sequences of (B-G) MDG1 retrotransposons, and (H) the enveloped insect-ERV ZAM as a control. Left panels: ovaries, comparing control (*Tj>Gal4, arr2-RNAi*, top) and somatic piRNA pathway knockdown (*Tj>Gal4, vret-RNAi*, bottom). Right panels: OSCs, comparing control (treated with siRNA targeting GFP) and piRNA pathway knockdown (treated with siRNA targeting Piwi). *Stalker1* appears to lack a premature transcription termination site, based on the 3' end data and sequence analysis. For *412* the ovaries samples are shown in main Figure 3B. The Y-axis indicates the number of reads.

bioRxiv preprint doi: https://doi.org/10.1101/2025.03.14.642691; this version posted March 16, 2025. The copyright holder for this preprint (which was not certified by peer review) is the author/funder, who has granted bioRxiv a license to display the preprint in perpetuity. It is made available under aCC-BY 4.0 International license.

A

Number of peptides detected in mass-spec	OSCs						Ovaries	
	Control #1	Control #2	Control #3	siPIWI #1	siPIWI #2	siPIWI #3	Control	Somatic knockdown
412 sORF1	-	-	-	3	2	3	-	1
412 sORF2	-	-	-	1	1	1	-	-
412 Gag	-	-	-	12	6	11	-	3
Mdg1 sORF1	-	-	-	2	1	-	-	-
Mdg1 sORF2	-	-	-	3	2	3	-	-
Mdg1 Gag	-	-	-	10	11	10	-	-
Mdg1 Pol	-	-	-	1	5	4	-	-
Blood sORF1	-	2	-	5	3	4	-	2
Blood Gag	1	-	-	12	10	8	-	3
Blood Pol	-	-	-	1	1	2	-	-
Stalker1 sORF2	-	-	-	2	2	2	-	-
Stalker2 sORF1	-	-	-	3	1	2	-	-
Stalker2 Gag	-	-	-	2	3	3	-	-
Tabor Gag	-	-	-	6	9	8	-	-

B

412 sORF1

MGKHRGGLKTKLNKRIAHVRWLIYGQQTPPVWSKALKLHAELDHLLQYQQELKTHKLEK
THSDNIKNIPTINEVPKYQACPAPAPALTKPKKSCPPGCEGIWSPHCLGTCERPSTSSAAKK

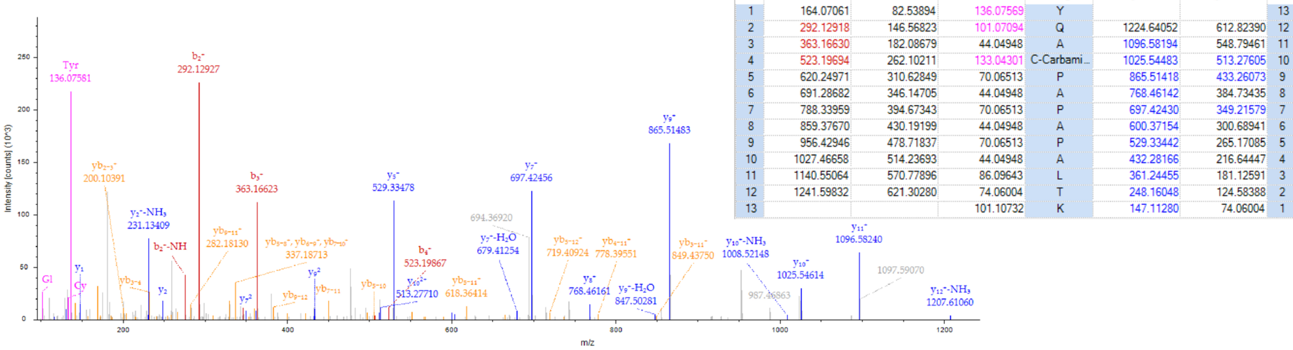

YQACPAPAPALTK

#1	b+	b2+	Immonium	Seq.	y+	y2+	#2
1	164.07061	82.53894	136.07569	Y			13
2	292.12918	146.56823	101.07094	Q	1224.64052	612.82390	12
3	363.16630	182.08679	44.04948	A	1096.58194	548.79461	11
4	523.19694	262.10211	133.04301	C-Carbami...	1025.54483	513.27605	10
5	620.24971	310.62849	70.06513	P	865.51418	433.26073	9
6	691.28682	346.14705	44.04948	A	768.46142	384.73435	8
7	788.33959	394.67343	70.06513	P	697.42430	349.21579	7
8	859.37670	430.19199	44.04948	A	600.37154	300.68941	6
9	956.42946	478.71837	70.06513	P	529.33442	265.17085	5
10	1027.46658	514.23693	44.04948	A	432.28166	216.64447	4
11	1140.55064	570.77896	86.09643	L	361.24455	181.12591	3
12	1241.59832	621.30280	74.06004	T	248.16048	124.58388	2
13			101.10732	K	147.11280	74.06004	1

412 sORF2

MGLIDTKTVDSTANVINKVEVQNADLFWITIILIVIALIMVSNALIKIYKLHNKCLKKRYRSTANGIDNI

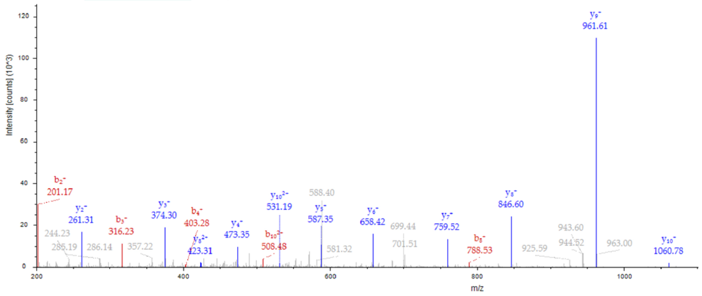

TVDSTANVINK

#1	b+	b2+	Seq.	y+	y2+	#2
1	102.05496	51.53112	T			11
2	201.12337	101.06532	V	1060.56331	530.78530	10
3	316.15031	158.57879	D	961.49490	481.25109	9
4	403.18234	202.09481	S	846.46796	423.73762	8
5	504.23002	252.61865	T	759.43593	380.22160	7
6	575.26713	288.13720	A	658.38825	329.69776	6
7	689.31006	345.15867	N	587.35114	294.17921	5
8	788.37847	394.69288	V	473.30821	237.15774	4
9	901.46254	451.23491	I	374.23980	187.62354	3
10	1015.50546	508.25637	N	261.15573	131.08150	2
11			K	147.11280	74.06004	1

C

Mdg1 sORF1

MGKNRGGVRHNIKKRIAHIKWLLYTNTPPPQWSKAQKLQAELDHCVEYLKKKIPTTRAHSENQIKSLTINKTPTPNPKSLPVFKKRCPNDCEGPLFTPHCEHTCRHCSSTT

Mdg1 sORF2

MGWFGSDDSQTKDNTANVVNNLKIVDHTDDIQSLWLLLLIMTIVTVAQFILTLYVKHNKIIKRRYIGKAENSLDKI

Blood sORF1

MSTKQTFEHPAPVEQRDLPSIKEVIEVDPSAGPKPLTIQEYKARTAAREQPPKKKRGGRRIKLLSARRLNIELLKTATNEEDRQRYKERLAAINQQLRGAK

Stalker1 sORF2

MGWFSDSSEAKDNTANVVNNVKIIDHTDDINALWILLLIITIVLLLQFLLTIYVKHNKIIKRRYMNRANRLDQI

Stalker2 sORF1

MADEPQFANAQPQVQRDQPTLEEALRLNNADGPRPLTVAEYRARQEKKQLRKHKRSGRRIKLLQQRRLVKEMTQLAKEESARQRYQERLEAIEQELRQSAKTRKRAA

bioRxiv preprint doi: https://doi.org/10.1101/2025.03.14.642691; this version posted March 16, 2025. The copyright holder for this preprint (which was not certified by peer review) is the author/funder, who has granted bioRxiv a license to display the preprint in perpetuity. It is made available under aCC-BY 4.0 International license.

Supplementary Figure 5: Untargeted proteomics evidence for translation of MDG1 retrotransposon ORFs. **(A)** Mass-spectrometry detection of peptides corresponding to sORF1 (purple), sORF2 (cyan) and Gag (orange) sequences of MDG1 LTR retrotransposons in control OSCs (treated with siRNA targeting GFP), piRNA pathway knockdown OSCs (treated with siRNA targeting Piwi), control ovaries (*Tj>Gal4, arr2-RNAi*) and somatic piRNA pathway knockdown ovaries (*Tj>Gal4, vret-RNAi*). **(B)** Two representative fragment spectra of detected peptides supporting the presence of 412 sORF1 in somatic piRNA pathway knockdown ovaries and sORF2 in piRNA pathway knockdown OSCs. **(C)** Sequences of sORF1 and sORF2 peptides detected in mass-spec, supporting the translation of these proteins from MDG1 retrotransposons.

bioRxiv preprint doi: https://doi.org/10.1101/2025.03.14.642691; this version posted March 16, 2025. The copyright holder for this preprint (which was not certified by peer review) is the author/funder, who has granted bioRxiv a license to display the preprint in perpetuity. It is made available under aCC-BY 4.0 International license.

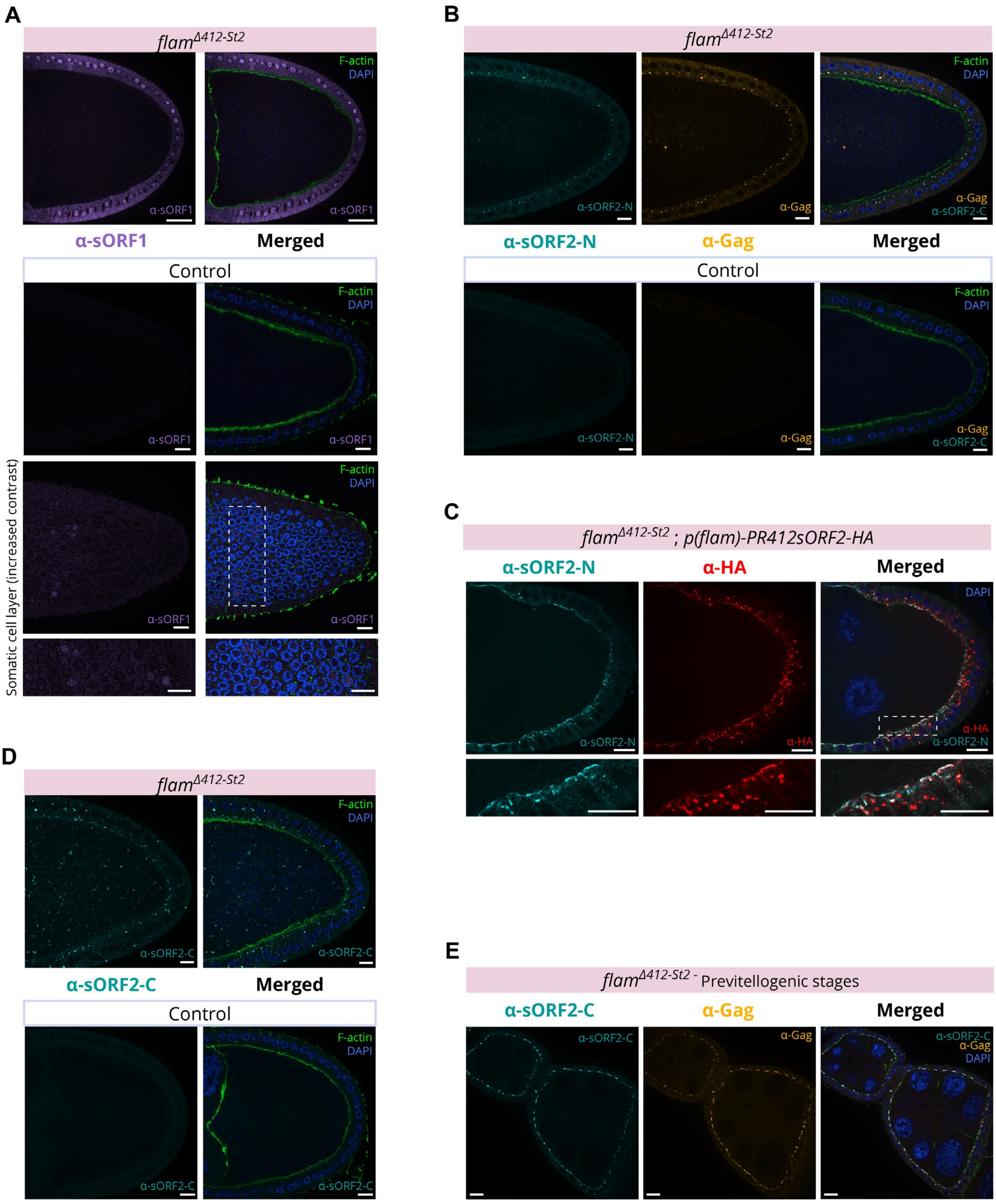

bioRxiv preprint doi: https://doi.org/10.1101/2025.03.14.642691; this version posted March 16, 2025. The copyright holder for this preprint (which was not certified by peer review) is the author/funder, who has granted bioRxiv a license to display the preprint in perpetuity. It is made available under aCC-BY 4.0 International license.

Supplementary Figure 6: Antibodies targeting sORF1 and sORF2 are specific and support distinct cellular localizations. **(A)** Whole mount immunofluorescence of *flam*$^{\Delta412\text{-}St2}$ ovaries (top) and control ovaries (bottom) with α-sORF1 antibody (purple). A weak nuclear staining of sORF1 is visible in control ovaries with increased contrast settings, consistent with RNA-seq and mass-spec data (bottom panels). **(B)** Whole mount immunofluorescence of *flam*$^{\Delta412\text{-}St2}$ ovaries (top) and control ovaries (bottom) stained with α-sORF2-N (cyan) and α-Gag (orange). **(C)** Whole mount immunofluorescence of *flam*$^{\Delta412\text{-}St2}$ ovaries expressing a C-terminal HA-tagged *412 sORF2* transgene under a *flamenco* promoter, stained with α-sORF2-N (cyan) and α-HA (red), showing good co-localization (white) of the two antibodies on the apical somatic membranes. **(D)** Whole mount immunofluorescence of *flam*$^{\Delta412\text{-}St2}$ ovaries (top) and control ovaries (bottom) with α-sORF2-C (cyan). **(E)** Co-localization of α-sORF2-C (cyan) and α-Gag (orange) on the apical somatic cell membranes in previtellogenic stages. For (A-B, D-E) F-actin is labelled with phalloidin (green) to demarcate cortical actin near the plasma membrane. Scale bars (A-E): 10 µm.

bioRxiv preprint doi: https://doi.org/10.1101/2025.03.14.642691; this version posted March 16, 2025. The copyright holder for this preprint (which was not certified by peer review) is the author/funder, who has granted bioRxiv a license to display the preprint in perpetuity. It is made available under aCC-BY 4.0 International license.

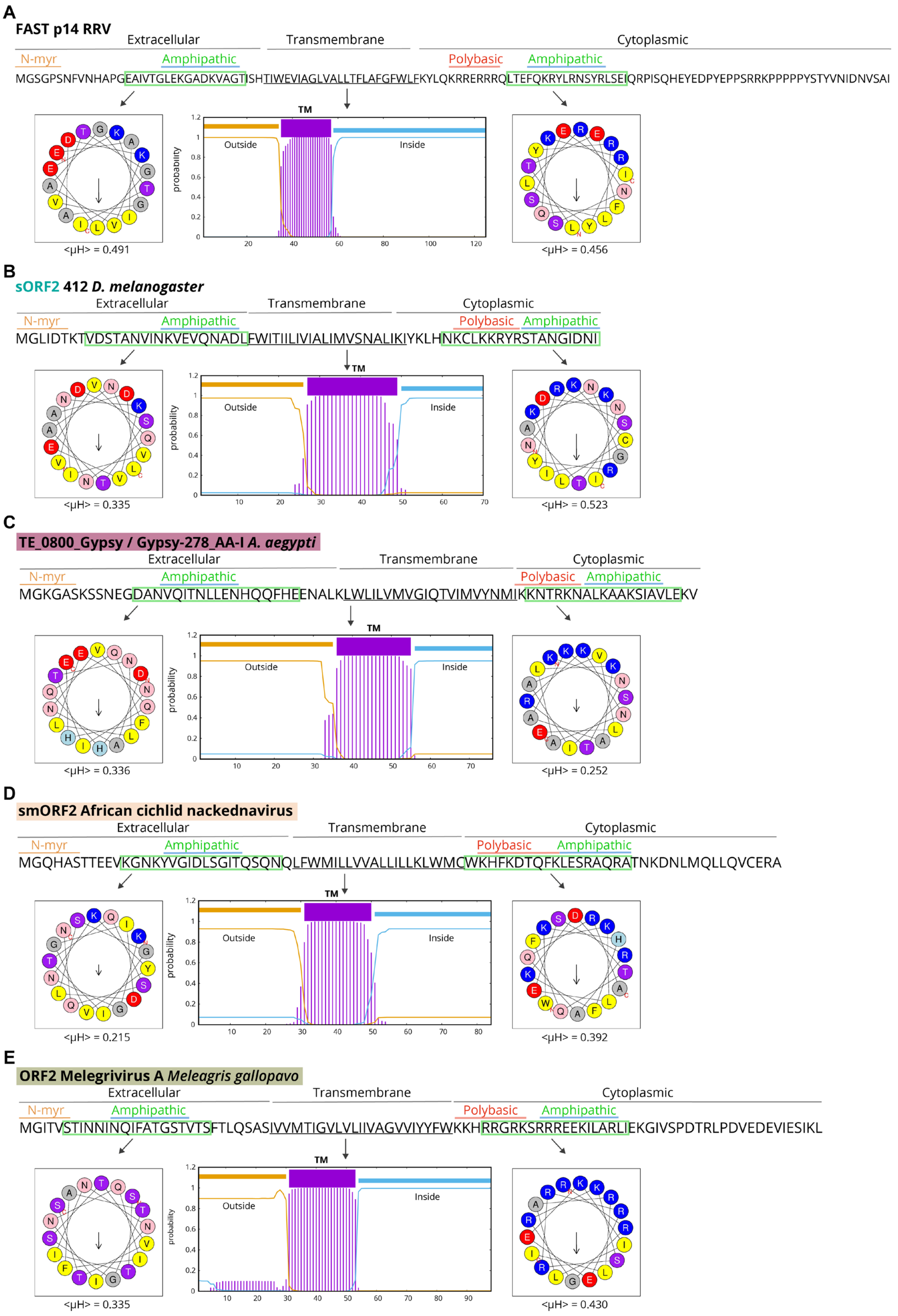

bioRxiv preprint doi: https://doi.org/10.1101/2025.03.14.642691; this version posted March 16, 2025. The copyright holder for this preprint (which was not certified by peer review) is the author/funder, who has granted bioRxiv a license to display the preprint in perpetuity. It is made available under aCC-BY 4.0 International license.

Supplementary Figure 7: Transmembrane and amphipathic domains in newly discovered fusogen-like proteins. Predictions for amphipathic helices using Heliquest and for transmembrane domains using TMHMM (see Materials & Methods) in (A) known FAST protein p14 from reptilian orthoreovirus (Genbank AAP03134.1), (B) sORF2 from *412* LTR retrotransposon (Genbank X04132), (C) Newly identified ORF within TE_0800_Gypsy/Gypsy-278 from *A. aegyptii* genome (Genbank GCF_002204515.2), (D) smORF2 from African cichlid nackednavirus (Genbank MH158727) and (E) ORF2 from turkey-infecting Melegrivirus A (Genbank KF961188). Helical projections and the hydrophobic moment values <μH> shows various degrees of partitioning of hydrophobic residues (yellow) and polar/charged residues (blue, red, purple, pink) on opposite sides of the helix, corresponding to amphipathic helices.

bioRxiv preprint doi: https://doi.org/10.1101/2025.03.14.642691; this version posted March 16, 2025. The copyright holder for this preprint (which was not certified by peer review) is the author/funder, who has granted bioRxiv a license to display the preprint in perpetuity. It is made available under aCC-BY 4.0 International license.

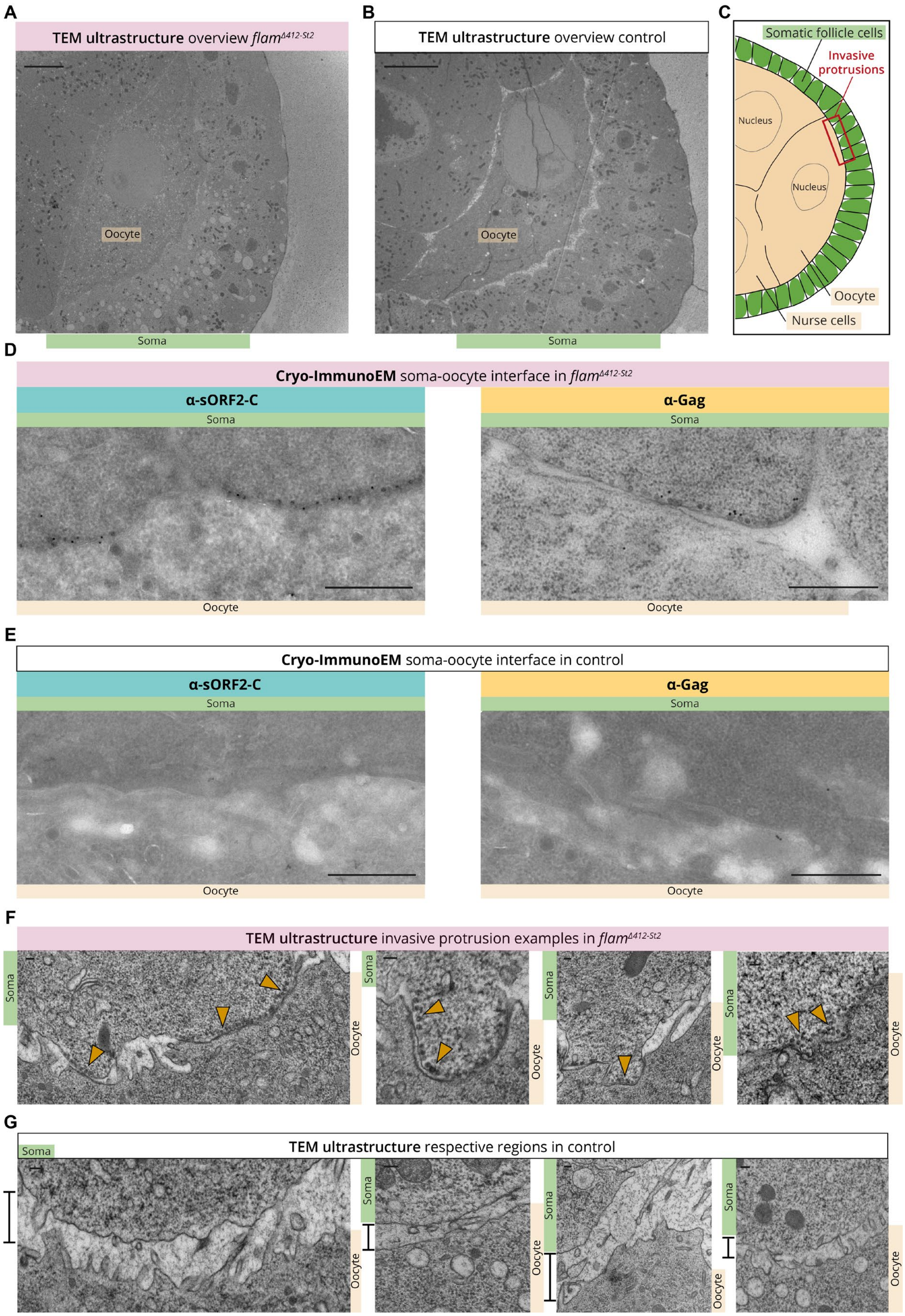

bioRxiv preprint doi: https://doi.org/10.1101/2025.03.14.642691; this version posted March 16, 2025. The copyright holder for this preprint (which was not certified by peer review) is the author/funder, who has granted bioRxiv a license to display the preprint in perpetuity. It is made available under aCC-BY 4.0 International license.

Supplementary Figure 8: Characterization and validation of observed capsids and protrusions in *flam*$^{\Delta412\text{-}St2}$ follicles. **(A-C)** Overview of region of interest in TEM ultrastructure studies. (A) Stage 7 *flam*$^{\Delta412\text{-}St2}$ follicle does not show any major morphological defects compared to (B) control follicle of the same stage. For (A-B) Scale bar: 5 µm (C) Cartoon of characteristic region (boxed red rectangle) for observed invasive protrusions in the soma-oocyte interface of *flam*$^{\Delta412\text{-}St2}$ follicles. **(D)** Cryo-immunoEM of *flam*$^{\Delta412\text{-}St2}$ stage 7 follicle using α-sORF2-C antibody (left image, gold particles as black dots) and α-Gag (right image, gold particles as black dots), in proximity to accumulated capsids on the apical membranes of somatic follicle cells. **(E)** Same for (D), only for control ovaries. For (D-E) Scale bar: 500 nm. **(F-G)** Observation of soma-oocyte interface in ultrastructure of (F) *flam*$^{\Delta412\text{-}St2}$ follicles and (G) control follicles. Notice the appearance of capsid-filled invasive protrusions (orange arrowheads) which are in close contact with the oocyte membrane in *flam*$^{\Delta412\text{-}St2}$ follicles, as opposed to control follicles lacking such structures and a larger distance between soma and oocyte membranes (black bar). For (F-G) Scale bar: 100 nm.

bioRxiv preprint doi: https://doi.org/10.1101/2025.03.14.642691; this version posted March 16, 2025. The copyright holder for this preprint (which was not certified by peer review) is the author/funder, who has granted bioRxiv a license to display the preprint in perpetuity. It is made available under aCC-BY 4.0 International license.

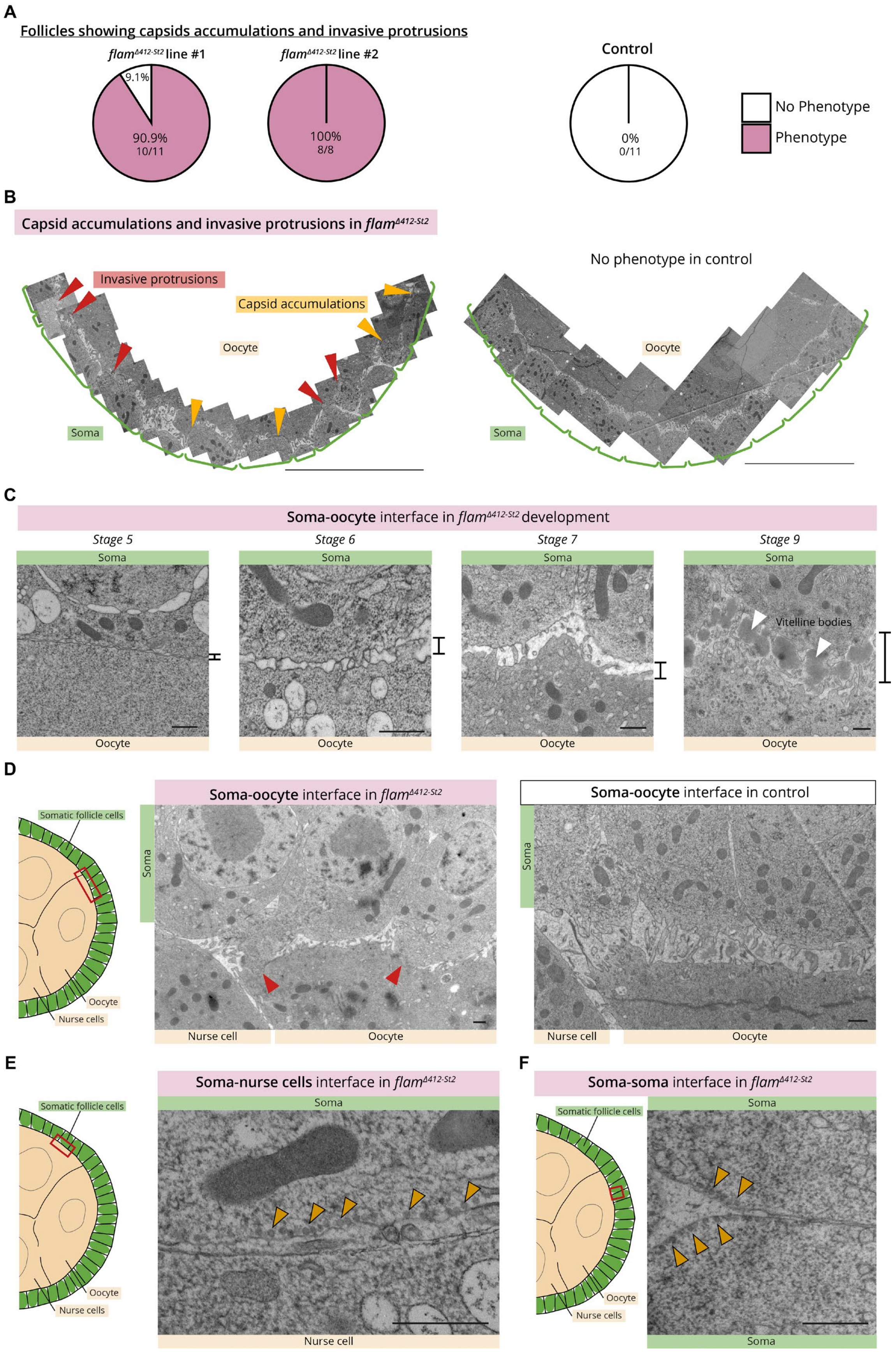

bioRxiv preprint doi: https://doi.org/10.1101/2025.03.14.642691; this version posted March 16, 2025. The copyright holder for this preprint (which was not certified by peer review) is the author/funder, who has granted bioRxiv a license to display the preprint in perpetuity. It is made available under aCC-BY 4.0 International license.

Supplementary Figure 9: Invasive protrusions form exclusively in the soma-oocyte interface of *flam*$^{\Delta412\text{-}St2}$ follicles during a developmental time window. (A) Quantification of follicles exhibiting capsid accumulations and invasive protrusions as observed in TEM ultrastructure of two independent mutant *flam*$^{\Delta412\text{-}St2}$ lines and control ovaries. **(B)** Spatial distribution of capsid accumulations (orange arrowheads) and invasive protrusions (red arrowheads) along the apical membranes of somatic follicle cells (green brackets) facing the oocyte. Images were manually stitched for representative *flam*$^{\Delta412\text{-}St2}$ and control follicles. Scale bar: 10 µm. High-resolution images are available in Supplemental Data files S1 and S2. **(C)** Soma-oocyte interface of *flam*$^{\Delta412\text{-}St2}$ follicles at different developmental stages. Notice in stage 5 the absence of microvilli and close association of somatic and oocyte membranes. As development progresses, somatic and oocyte membranes separate but maintain somatic microvilli across the perivitelline space, until deposited vitelline bodies coalesce to form the protective layer of the egg. **(D)** Invasive protrusions (red arrows) in the soma-oocyte interface of *flam*$^{\Delta412\text{-}St2}$ stage 7 follicle (left), and the respective area in a control follicle of similar developmental stage (right). Notice the reduced spacing between somatic and oocyte membranes in the *flam*$^{\Delta412\text{-}St2}$ follicle. **(E)** Capsid accumulation (orange arrowheads pointing to several capsids) along the apical membrane of a somatic cell facing the nurse cells. **(F)** Capsid accumulations along the lateral membranes of two somatic cells, close to the apical side. For (C-F) Scale bar: 500 nm.

bioRxiv preprint doi: https://doi.org/10.1101/2025.03.14.642691; this version posted March 16, 2025. The copyright holder for this preprint (which was not certified by peer review) is the author/funder, who has granted bioRxiv a license to display the preprint in perpetuity. It is made available under aCC-BY 4.0 International license.

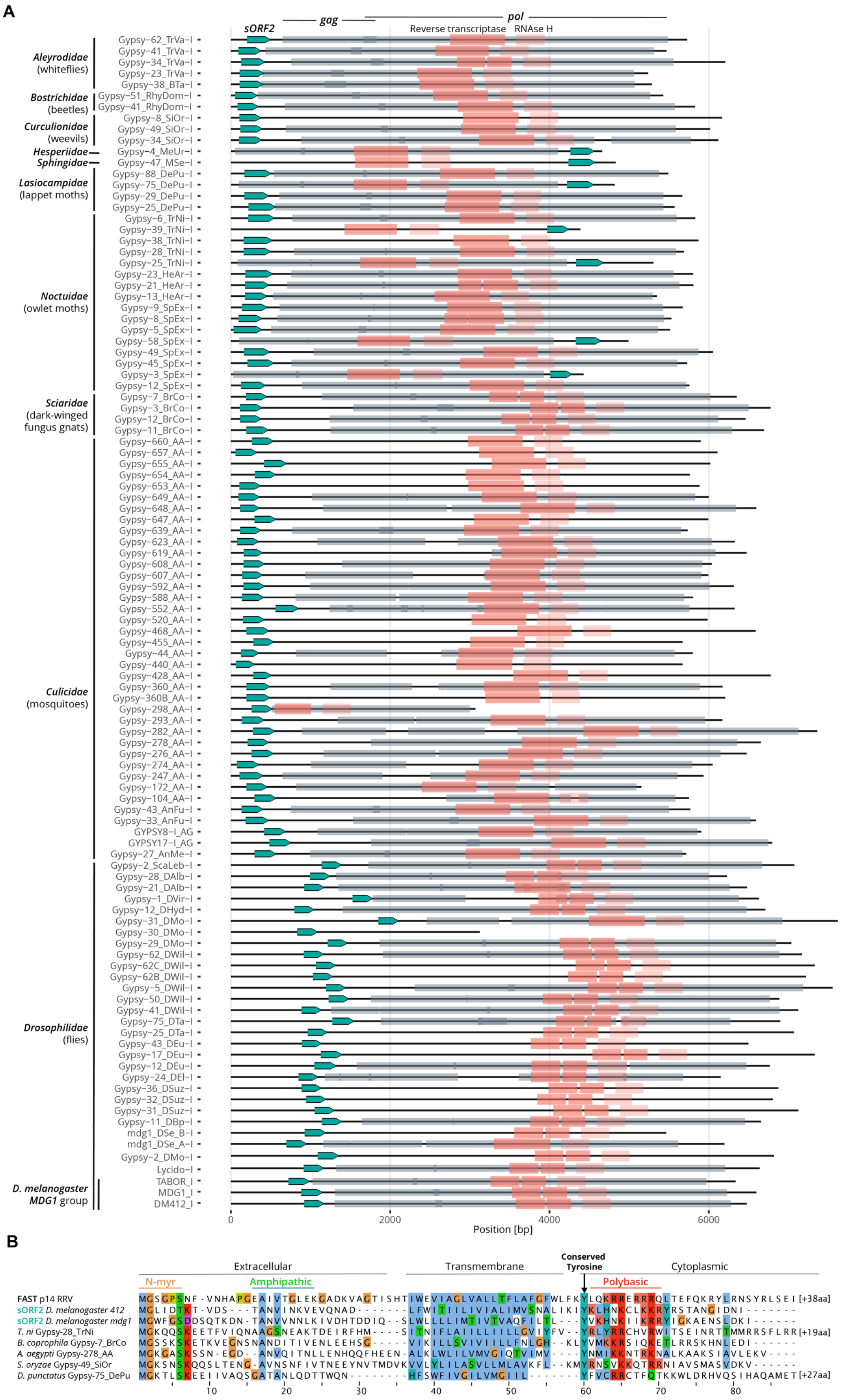

bioRxiv preprint doi: https://doi.org/10.1101/2025.03.14.642691; this version posted March 16, 2025. The copyright holder for this preprint (which was not certified by peer review) is the author/funder, who has granted bioRxiv a license to display the preprint in perpetuity. It is made available under aCC-BY 4.0 International license.

Supplementary Figure 10: *sORF2/FAST*-like genes are encoded within *Metaviridae* in insect genomes. (A) Visual representation of internal consensus sequences from Repbase of all 105 identified *Gypsy* LTR retrotransposons with an *sORF2/FAST*-like gene (cyan arrow), across different insect families. Annotated ORFs from Repbase shown in grey boxes, likely corresponding to *gag* and *pol* ORFs. Identified regions with conserved domains reverse-transcriptase (dark pink) and RNAse H (light pink) support *pol* annotation. *LTR* regions are not shown. **(B)** Multiple sequence alignment of a known FAST protein p14, newly found sORF2 proteins from *D. melanogaster* and 5 representative sequences of sORF2/FAST-like proteins from insect *Metaviridae*. Notice conservation of all known structural features for FAST proteins and a newly identified Tyr residue just downstream of the TM.

bioRxiv preprint doi: https://doi.org/10.1101/2025.03.14.642691; this version posted March 16, 2025. The copyright holder for this preprint (which was not certified by peer review) is the author/funder, who has granted bioRxiv a license to display the preprint in perpetuity. It is made available under aCC-BY 4.0 International license.

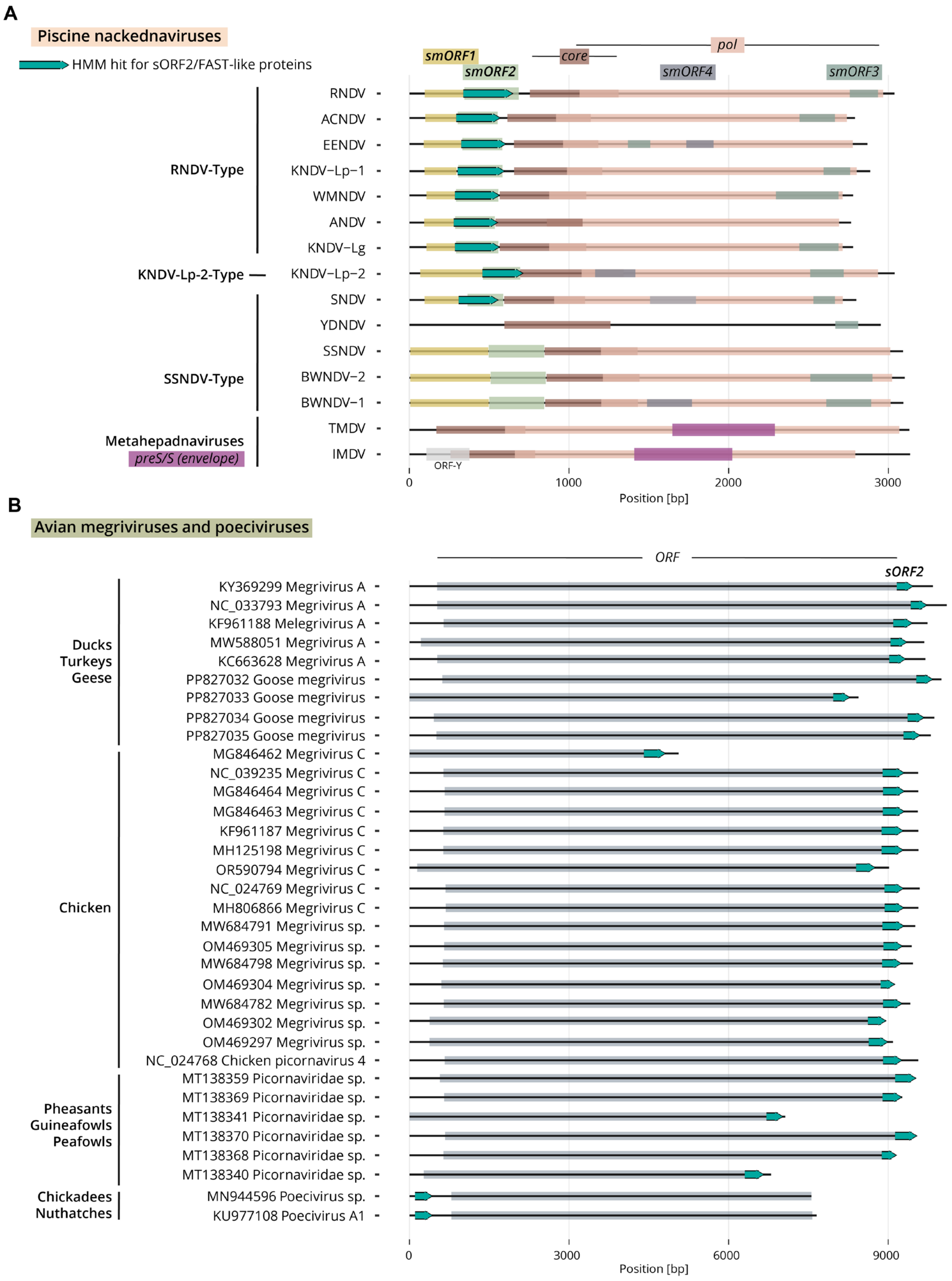

bioRxiv preprint doi: https://doi.org/10.1101/2025.03.14.642691; this version posted March 16, 2025. The copyright holder for this preprint (which was not certified by peer review) is the author/funder, who has granted bioRxiv a license to display the preprint in perpetuity. It is made available under aCC-BY 4.0 International license.

Supplementary Figure 11: *sORF2/FAST*-like genes are encoded as separate ORFs within non-enveloped viral genomes. **(A)** Visual representation of assembled and annotated nackednavirus genomes according to (Lauber et al., 2017), with the putative smORF2 genes that were found by HMM-based search (cyan arrows). All other annotated ORFs are in colored boxes. SNDV and YDNDV have partial smORF2 sequences assembled. TMDV and IMDV are shown for comparison, with the annotated *preS/S* gene in violet **(B)** Visual representation of assembled and annotated megriviruses and poeciviruses from NCBI viral genomes (1,500-20,000 nt), with the putative *sORF2/FAST-like* ORF2 genes that were found by HMM-based search (cyan arrows). The hosts from which the viruses were sampled are noted on the left.

bioRxiv preprint doi: https://doi.org/10.1101/2025.03.14.642691; this version posted March 16, 2025. The copyright holder for this preprint (which was not certified by peer review) is the author/funder, who has granted bioRxiv a license to display the preprint in perpetuity. It is made available under aCC-BY 4.0 International license.

Other supplementary materials for this manuscript:

Supplementary Table S1: *sORF2/FAST*-like sequences identified in this study. The different tabs list sORF2/FAST-like sequences found in**:** (A) *Gypsy* LTR retrotransposon of insects. (B) *D. melanogaster*. (C) *A. aegyptii*. (D) *S. oryzae*. (E) Nackednaviruses. (F) Picornaviruses.

Supplementary Table S2: Fly genotypes, smFISH probes and siRNA sequences used in this study.

Supplementary Movie S1: Electron tomography of an invasive protrusion in a *flam*$^{\Delta 412\text{-}St2}$ follicle. The movie shows consecutive Z-sections from a tilt-series tomogram and a 3D rendering of an invasive protrusion containing retrotransposon capsids (pink), the somatic follicle cell membrane (green) and oocyte membrane (beige). Notice that the somatic and oocyte membranes are in extremely proximity, specifically at the site of the protrusion.

Data S1: High resolution spatial localization of capsid accumulations and invasive protrusions at the oocyte-soma interface in *flam*$^{\Delta 412\text{-}St2}$ follicle (same image as fig. S9, left)

Data S2: High resolution oocyte-soma interface in control follicle (same image as fig. S9, right)
